# The Pel polysaccharide modulates interspecies interactions between *Pseudomonas aeruginosa* and *Mycobacterium abscessus*

**DOI:** 10.1128/msystems.00270-26

**Published:** 2026-05-18

**Authors:** Melissa S. McDaniel, Sara E. Edmonds, Evani N. Patel, Joshua J. Baty, Jessica A. Scoffield

**Affiliations:** 1 Radboud University Medical Center 6034, Nijmegen, the Netherlands; 2 Department of Microbiology, University of Alabama at Birmingham 318277, Birmingham, Alabama, USA; 3 School of Biological Sciences, Georgia Institute of Technology 123387, Atlanta, Georgia, USA; National Cancer Institute Center for Cancer Research, Bethesda, Maryland, USA

**Keywords:** polymicrobial, biofilm, *P. aeruginosa*, NTM

## Abstract

Modulator therapies have improved outcomes for people with cystic fibrosis (pwCF), and currently, more than 50% of pwCF are over the age of 18. This has resulted in an increased prevalence of atypical pathogens, including non-tuberculous mycobacteria (NTM). CF-isolation rates of NTM and *Pseudomonas aeruginosa* are high, and those co-colonized have worse clinical outcomes. We therefore investigated the behavior of these two organisms in a dual-species biofilm. We found that co-culture of *Mycobacterium abscessus* (MAB) promoted biofilm formation by *P. aeruginosa*. Confocal imaging revealed changes in biomass and structural organization of the *P. aeruginosa* biofilm during co-culture with MAB. DNase treatment slightly decreased dual-species biofilm, but biofilm formation was abrogated in a Pel-deficient mutant of *P. aeruginosa*. Moreover, MAB stimulated the overproduction of Pel polysaccharide in dual-species biofilms with *P. aeruginosa*. Furthermore, dual-species cultures promoted tolerance of *P. aeruginosa* to tobramycin treatment. Finally, transcriptomics analysis revealed that MAB alters the expression of genes that impact biofilm formation in *P. aeruginosa*. Overall, our findings highlight a Pel-dependent interaction between *P. aeruginosa* and *M. abscessus* that may result in bacterial persistence for pwCF during antibiotic therapy.

**IMPORTANCE:**

Advances in treatments for cystic fibrosis have increased the incidence of “non-typical” infections, including those caused by non-tuberculous mycobacteria (NTM). NTM are often found in the lungs alongside the pathogen *Pseudomonas aeruginosa*, and patients infected with both tend to have worse clinical outcomes. In this study, we examined how these two organisms interact under biofilm conditions, an important lifestyle adaptation in chronic pulmonary infections. We found that when grown together, *Mycobacterium abscessus* enhanced biofilm formation by *P. aeruginosa* and reduced the effectiveness of antibiotic treatment. Our findings reveal a Pel-dependent interaction between two important lung pathogens that may contribute to more severe infections and poorer treatment outcomes in people with cystic fibrosis or other chronic lung diseases. Understanding these mechanisms opens new opportunities for developing strategies to combat these complex infections.

## INTRODUCTION

Cystic fibrosis (CF) is a genetic disorder caused by mutations in the cystic fibrosis transmembrane conductance regulator (CFTR). Pulmonary manifestations are characterized by hyperinflammation, muco-obstruction, and decreased mucus clearance, coupled with innate immune dysfunction (1–3). These factors lead to chronic pulmonary bacterial infections that cannot be resolved, despite dramatic inflammation of the airways. Prior to the advent of CFTR modulator therapies, these infections and the associated decline in lung function were the primary drivers of morbidity and mortality in persons with CF (pwCF), and although these treatments have gone very far in improving lung function and quality of life, they have failed to completely eradicate bacterial infections in pwCF (4–8). Furthermore, the increasing age of pwCF and the long-term use of antibiotics to treat infections in this population have led to the emergence of non-traditional and antibiotic-resistant CF pathogens, including non-tuberculous mycobacteria (NTM) (9).

NTM are a diverse group of bacteria commonly isolated from environmental sources that can act as opportunistic pathogens, causing a variety of disease manifestations within chronic respiratory infections (10, 11). In pwCF, *Mycobacterium abscessus* (MAB) and *Mycobacterium avium* complexes (MAC) are the most prevalent NTM species, with MAB colonization being associated with a more severe decline in lung function compared to MAC or other traditional CF pathogens (12, 13). Unlike infections with *Mycobacterium tuberculosis,* NTM infections may exist both intracellularly (with macrophages as the primary reservoir) and extracellularly, likely as a biofilm within the lung (14, 15). This makes the ability to persist within a biofilm potentially important for the virulence of this organism, both for the mechanism of initial acquisition and for persistence in the lung (14–18). MAB also has two known morphotypes, with the smooth (S) morphotype indicating production of glycopeptidolipids and the rough (R) morphotype associated with the loss of their production (19, 20). The rough morphotype is known to emerge over the course of human disease, increase resistance to phagocytosis, and be associated with a more virulent infection (19, 21–27).

Thickened mucus and a decrease in mucociliary clearance predispose the CF lung to colonization with pathogens in the form of a chronic, complex polymicrobial biofilm, in which the predominant pathogen is *Pseudomonas aeruginosa* (*Pa*). This highly virulent organism is found in over half of pwCF and is known to interact with many CF pathogens during co-infection to alter infection dynamics (28–43). *P. aeruginosa* also displays well-studied adaptations specific to the CF lung, including the transition from early exopolysaccharides, such as Pel and Psl, to the late-stage exopolysaccharide alginate. Each of these exopolysaccharides has been shown to shape interactions with other organisms, particularly with the common CF pathogen *Staphylococcus aureus* (41, 44–50). The acquisition of NTM is not well correlated with the presence of *Pa,* although they can be co-isolated at relatively high rates (51–53). Importantly, non-CF bronchiectasis patients co-infected with NTM and *P. aeruginosa* are well documented to have a steeper rate of lung decline than those patients infected with either alone, indicating synergy between these two organisms in terms of virulence (54–56). In further support of this, previous publications that focused on dual-species interactions between *P. aeruginosa* and MAB have indicated that co-culture can provide a benefit to both species. MAB and *P. aeruginosa* can form dual-species biofilms, and co-culture in the presence of antibiotic treatment promotes MAB growth (57, 58). It has also been shown that MAB can directly degrade the PQS quorum-signaling molecule of *P. aeruginosa* via a dioxygenase (Aqd). However, these studies did not directly address the role of MAB on *P. aeruginosa* physiology and fitness (59–61).

Previous experimental and epidemiological studies indicate that interactions between *P. aeruginosa* and NTM contribute to the severity of infection. We therefore decided to investigate the behavior of these two organisms during co-culture in a dual-species biofilm. We found that despite rapid killing of MAB during co-culture, overall biofilm formation by *P. aeruginosa* increased in a manner dependent on both extracellular DNA (eDNA) and the Pel polysaccharide of *P. aeruginosa*. Co-culture of these two organisms also increased the tolerance of *P. aeruginosa* to tobramycin, an anti-pseudomonal antibiotic commonly used in a CF context. Ultimately, these results indicate that dual-species interactions between *P. aeruginosa* and MAB may contribute to an increase in disease severity during pulmonary infection.

## RESULTS

### Co-culture of *P. aeruginosa* and MAB promotes biofilm formation

**Fig 1:**
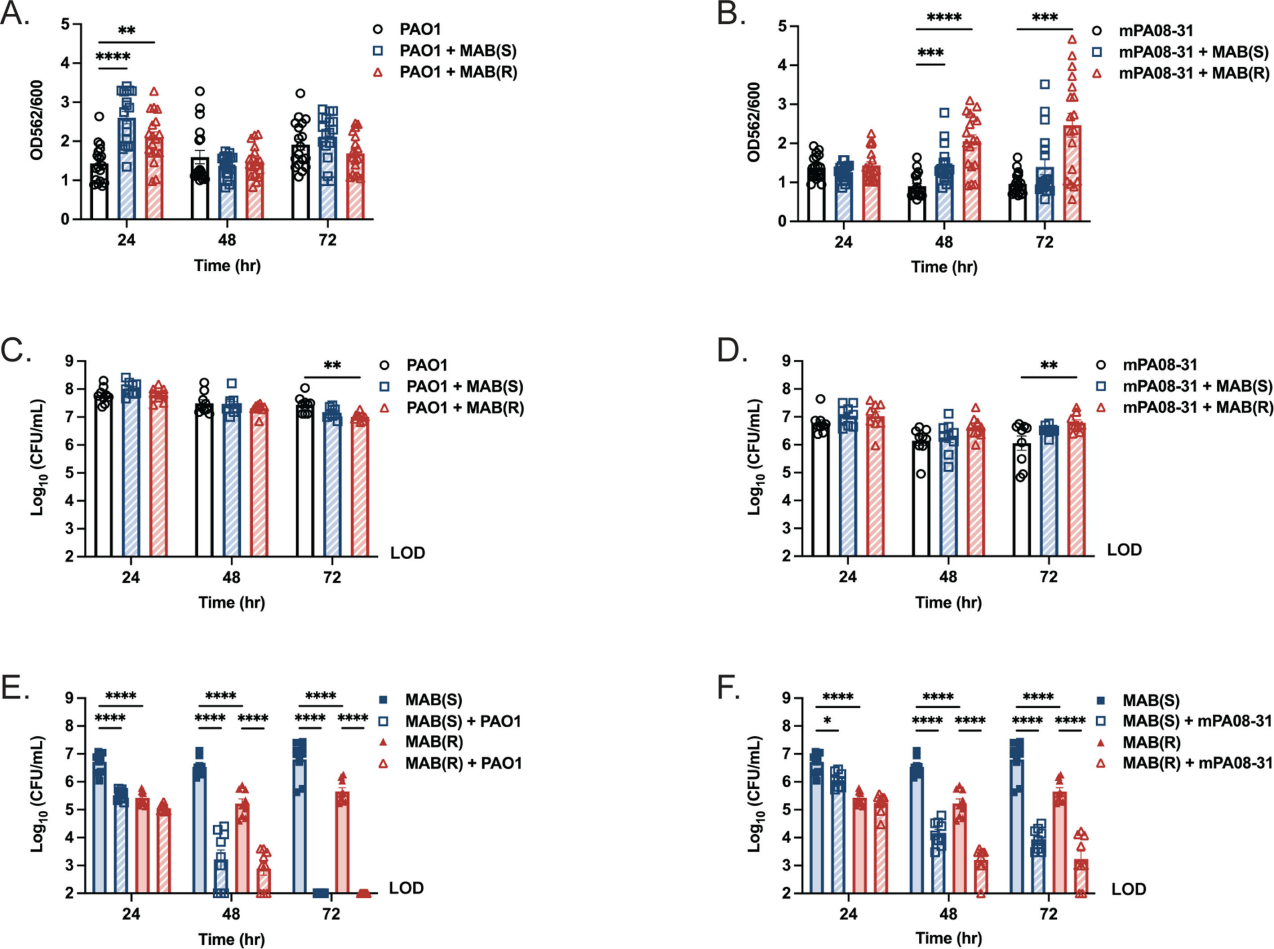
Co-culture of *P. aeruginosa* and MAB promotes biofilm formation. *M. abscessus* ATCC 19977 smooth and rough morphotypes were co-cultured with either of two *P. aeruginosa* strains (PAO1 or mPA08-31) in lysogeny broth medium in a 96-well plate for 24, 48, or 72 hours at 37°C. Biofilm biomass was then measured using crystal violet staining for (**A**) PAO1 and (**B**) mPA08-31 during single- and co-culture (*n* = 3 biological replicates and 6 technical replicates). Two-way ANOVA with Tukey’s multiple comparisons test was performed. Viable colony-forming units of *P. aeruginosa* (**C**) PAO1 and (**D**) mPA08-31 during single- and co-culture and of *M. abscessus* during single- and co-culture with (**E**) PAO1 and (**F**) mPA08-31 were measured (*n* = 3 biological replicates and 3 technical replicates). Two-way ANOVA with Tukey’s multiple comparisons test. *P* < 0.05 (*), *P* < 0.01 (**), *P* < 0.001 (***), *P* < 0.0001 (****).

To investigate the impact of dual-species co-culture on biofilm formation by *P. aeruginosa* and MAB, we first grew single- and dual-species biofilms with *P. aeruginosa* PAO1, a standard non-mucoid lab strain, or *P. aeruginosa* mPA08-31, a mucoid clinical strain, with *M. abscessus* smooth or rough morphotypes at varying time intervals. These were grown statically in flat-bottom 96-well plates without medium exchange. Biofilm biomass was measured via crystal violet assay and normalized to total growth. After 24 hours, biofilm biomass significantly increased when *P. aeruginosa* PAO1 was co-cultured with either the smooth or rough morphotypes of MAB (*P* < 0.0001, *P* = 0.0125). However, this phenotype was lost at the 48- and 72-hour time points (Fig. 1A). Conversely, when *P. aeruginosa* mPA08-31 was co-cultured with smooth or rough MAB, there was no difference between single- and dual-species biofilms at 24 hours post-inoculation. However, an increase in overall biomass was observed with the smooth and rough morphotype at 48 hours post-inoculation (*P* = 0.0032, *P* < 0.0001) and with only the rough morphotype at 72 hours post-inoculation (*P* = 0.0012) (Fig. 1B). To determine which species was responsible for the increase in biofilm, we then measured viable colony-forming units (CFUs) of each species via a differential plating scheme. There was no significant increase in *P. aeruginosa* CFUs for either strain compared to single-species culture at any of the time points measured except for the 72-hour time point for mPA08-31 with the rough morphotype (*P =* 0.004) (Fig. 1C and D). During co-culture with either *P. aeruginosa* PAO1 or mPA08-31, viable MAB rapidly decreased, with an approximately 3-log drop in CFUs of MAB(S) by 48 hours post-infection. With PAO1, MAB was below 10^2^ CFU/mL (the limit of detection) by 72 hours (Fig. 1E), while ~10^4^ CFU/mL of MAB remained when co-cultured with mPA08-31 (Fig. 1F), possibly explaining differences in kinetics of the biofilm increase. To determine whether this phenomenon requires live MAB or is the result of a response to cellular components of MAB, we prepared heat-killed MAB of both morphotypes. We found that heat-killed MAB failed to induce an increase in biofilm formation for *P. aeruginosa* PAO1 or mPA08-31 (Fig. S1A and B).

**Fig 2:**
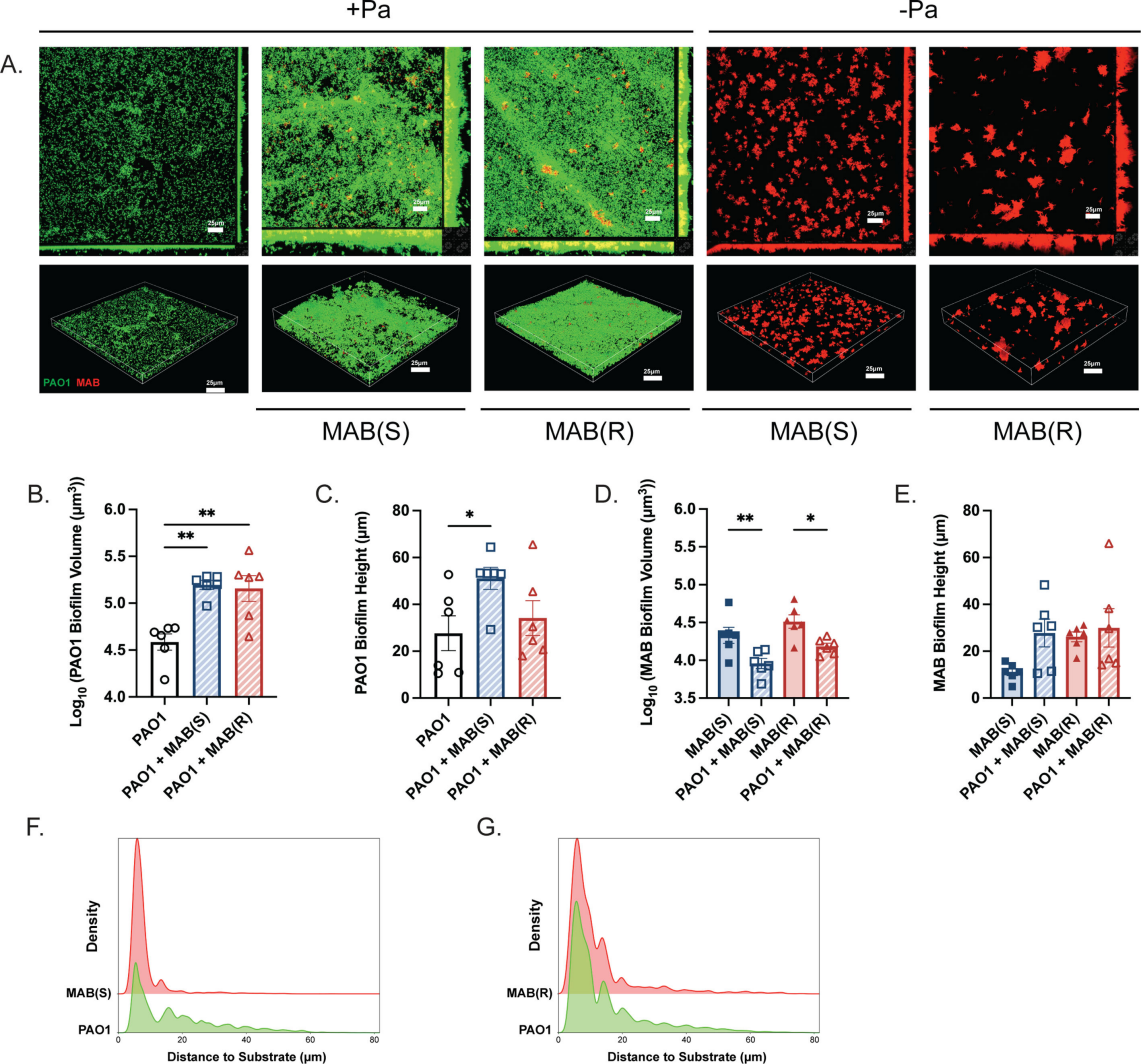
Imaging of dual-species *P. aeruginosa* and MAB biofilms shows changes in topology during co-culture. Fluorescently labeled *M. abscessus* ATCC 19977 smooth and rough morphotypes (mCherry+) were co-cultured with *P. aeruginosa* PAO1 (GFP+) in lysogeny broth medium in an 8-well μSlide for 24 hours at 37°C (*n* = 2 biological replicates and three technical replicates). (**A**) Structural composition of single- and dual-species biofilms was evaluated via confocal imaging at 40× magnification. (**B**) Volume and (**C**) height of *P. aeruginosa* PAO1 and (**D**) volume and (**E**) height of *M. abscessus* in single- and dual-species biofilms were quantified via BiofilmQ (62). One-way ANOVA with Dunnett’s multiple comparisons test. Histogram of pixel distribution of *P. aeruginosa* PAO1 and *M. abscessus* distance to substrate for (**F**) smooth and (**G**) rough morphotypes in dual-species biofilms (*n* = 2 biological replicates and 3 technical replicates). *P* < 0.05 (*), *P* < 0.01 (**).

Having observed an increase in biomass via crystal violet assay, we then investigated the biofilm structure using confocal microscopy. Single- and dual-species biofilms with *P. aeruginosa* PAO1^GFP+^ and MAB^mCherry+^ smooth or rough morphotypes were grown for 24, 48, or 72 hours, and the biofilm structure was subsequently imaged via confocal microscopy. As expected from the crystal violet assay, dual-species biofilms were visibly thicker than single-species biofilms with *P. aeruginosa* PAO1 alone at the 24-hour time point (Fig. 2A). Interestingly, we observed that MAB was localized primarily to the bottom of the biofilm, whereas *P. aeruginosa* can be found at the top, a phenomenon that has been seen by other groups (58). To confirm our visual findings, we quantified both biofilm height and volume for each species separately using BiofilmQ. We found that PAO1 biofilm volume was significantly increased during co-culture with both morphotypes of MAB at 24 hours (*P* = 0.0010, *P* = 0.0018), but not at subsequent time points (Fig. 2B; Fig. S2A). PAO1 biofilm height was only significantly increased with MAB(S) at the 24-hour time point (*P =* 0.0455) and only with MAB(R) at the 72-hour time point (*P* = 0.0044) (Fig. 2C; Fig. S2C). MAB biofilm volume trended toward a decrease in biomass compared to single-species biofilms at 24 hours but was significantly decreased at 48 and 72 hours (Fig. 2D; Fig. S2B). No significant changes in biofilm height were seen for MAB at the 24-hour time point (Fig. 2E; Fig. S2D). To quantify differences in localization within the dual-species biofilm, we plotted the distribution of distance to substrate for each cell within the biofilm. At 24 hours, the distribution of *P. aeruginosa* was notably right shifted, with a mean distance of 17.97 µm from the substrate, compared to the smooth morphotype of MAB with a mean distance of 8.096 µm (Fig. 2F; Fig. S2E and F). However, species were more evenly distributed when *P. aeruginosa* was grown with the rough morphotype of MAB (mean distance of 14.83 and 12.26 µm for PAO1 and MABR, respectively) (Fig. 2G) with similar trends at all time points tested (Fig. S2E and F). We repeated these experiments for *P. aeruginosa* mPA08-31. Similar to PAO1, we observed a visible increase in mPA08-31 biofilm thickness during co-culture. Biofilm volume was significantly increased for MAB(S) and MAB(R) at the 48-hour time point (*P =* 0.0181*, P =* 0.0173), and biofilm height was significantly increased when co-cultured with the rough morphotype at 72 hours (*P =* 0.0079) (Fig. S3A through C). In these biofilms, MAB volume decreased significantly during co-culture with both morphotypes at all time points tested (Fig. S3D). The relative distance to substrate was also like that seen in PAO1, though differences in localization were strongest at the 48- and 72-hour time points with the smooth morphotype (Fig. S3F and G).

Together, these findings demonstrate that co-culture with live MAB enhances *P. aeruginosa* biofilm biomass in a strain- and time-dependent manner, despite a pronounced decline in viable MAB over time. Confocal imaging revealed a morphotype-dependent stratification within these biofilms, with *P. aeruginosa* occupying the upper layers and the smooth morphotype of MAB sitting closer to the substratum.

### Extracellular DNA contributes to increased biofilm formation

**Fig 3:**
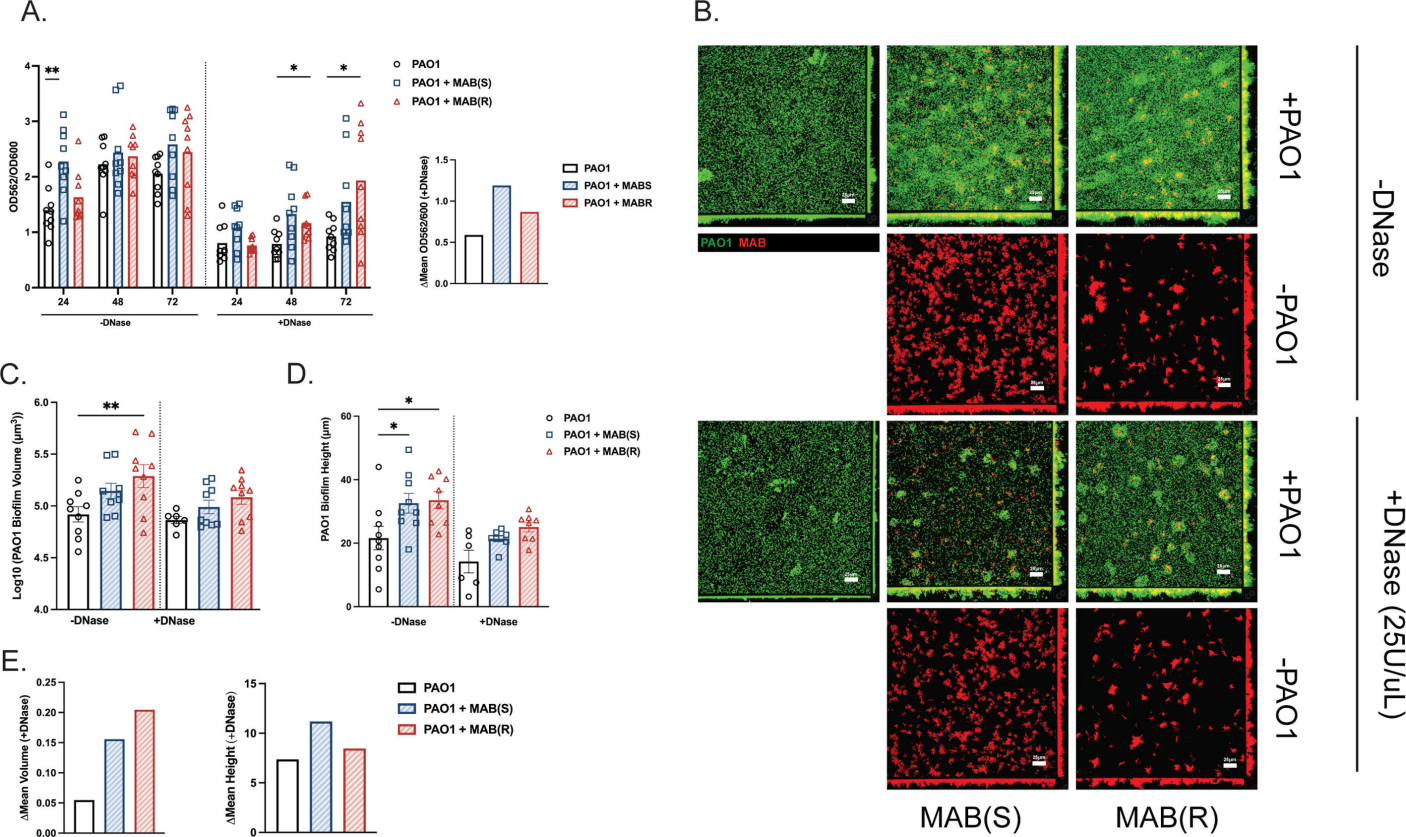
DNase treatment partially abrogates increased biofilm formation in the presence of MAB. *M. abscessus* ATCC 19977 smooth and rough morphotypes were co-cultured with *P. aeruginosa* strain PAO1 in lysogeny broth (LB) medium in a 96-well plate for 24, 48, or 72 hours at 37°C. Biofilms were then treated for 1 hour with DNase (25 U/mL) or phosphate-buffered saline. (**A**) Biofilm biomass measured using crystal violet staining during single- and co-culture (*n* = 3 biological replicates and 3 technical replicates). Two-way ANOVA with Tukey’s multiple comparisons test. Mean difference in OD_562/600_ with DNase treatment at 24 hours was also plotted. Fluorescently labeled *M. abscessus* ATCC 19977 smooth and rough morphotypes (mCherry+) were co-cultured with *P. aeruginosa* PAO1^GFP+^ in LB medium for 24 hours in an 8-well μSlide at 37°C (*n* = 2 biological replicates and 3 technical replicates). (**B**) Structural composition of single- and dual-species biofilms with and without DNase treatment was evaluated via confocal imaging at 40× magnification. Volume and height of (**C and D**) *P. aeruginosa* PAO1 was quantified via BiofilmQ (62) (*n* = 3 biological replicates and 3 technical replicates). One-way ANOVA with Šidák’s multiple comparisons test was performed. (**E**) Mean difference in biofilm volume and height between DNase-treated and untreated samples. *P* < 0.05 (*), *P* < 0.01 (**).

Due to the increase in overall biomass in our dual-species biofilms without an increase in viable CFUs, we hypothesized that extracellular DNA from dead MAB is responsible for the increased biomass in the dual-species biofilm. To test this, we grew single- and dual-species biofilms with or without DNase treatment and measured total biomass via crystal violet staining. For *P. aeruginosa* PAO1, crystal violet staining of dual-species biofilms without DNase treatment produced similar results to those in Fig. 1, with a significant increase in total biomass seen with dual-species biofilms only at the 24-hour time point and primarily for MAB(S) (*P =* 0.0069) (Fig. 3A). With DNase treatment, we observed reduced biomass at all time points, indicating the presence of eDNA in the biofilm. DNase treatment resulted in a loss of phenotype at the 24-hour time point, further evidenced by the greater difference in biofilm biomass in dual-species groups compared to the single-species group with DNase treatment (Fig. 3A). Little effect of DNase treatment was seen on either biofilm volume or height for MAB in single- or dual-species biofilms (Fig. S4). For mPA08-31, DNase treatment removed the increased biomass during co-culture at 48 hours, but biomass remained significantly increased for MAB(R) co-culture at 72 hours (*P* = 0.0026). The change in biomass with DNase treatment was higher in dual-species biofilms than in single-species biofilms for both the 48- and 72-hour time points (Fig. S5A and B).

To confirm the crystal violet results, we performed confocal microscopy on single- and dual-species biofilms of *P. aeruginosa* PAO1 after 24 hours and *P. aeruginosa* mPA08-31 after 72 hours, with or without a subsequent 1-hour DNase (25 U/mL) treatment. Without DNase treatment, PAO1 biofilms were thicker and denser with either MAB(S) or MAB(R) present. With DNase treatment, dual-species biofilms, although still visibly taller, appeared to have reduced density compared to those without DNase treatment (Fig. 3B). Similar trends were also seen for biofilms grown with mPA08-31, though there was perhaps less of an effect of DNase on biofilm composition for this strain (Fig. S5B). These results were confirmed through quantification, where PAO1 biofilm volume trended higher with MAB(S) and was significantly increased with MAB(R) without DNase treatment (*P* = 0.0090), and the significance of this increase was lost with DNase treatment (Fig. 3C). PAO1 biofilm height was significantly increased with either MAB(S) or MAB(R) without DNase treatment (*P* = 0.0233, *P =* 0.0154). This significance was lost following DNase treatment (Fig. 3D). For both biofilm volume and height, the difference after DNase treatment was greater in dual-species biofilms than with *P. aeruginosa* alone (Fig. 3E). For mPA08-31, the biofilm volume trended higher with MAB(S) and was significantly increased with MAB(R) without DNase treatment (*P* = 0.0032). The significance was lost with DNase treatment for MAB(R) but gained for MAB(S) (Fig. S5C). mPA08-31 biofilm height was significantly increased with either MAB(S) or MAB(R) without DNase treatment (*P* = 0.0012, *P* = 0.0204) but remained significant after DNase treatment (*P =* 0.0014, *P =* 0.0061) (Fig. S5D). Taken together, these data indicate that eDNA is a greater component of dual-species biofilms than single-species biofilms, particularly in PAO1.

### The Pel polysaccharide is required for increased biofilm biomass and is overproduced in dual biofilms

**Fig 4:**
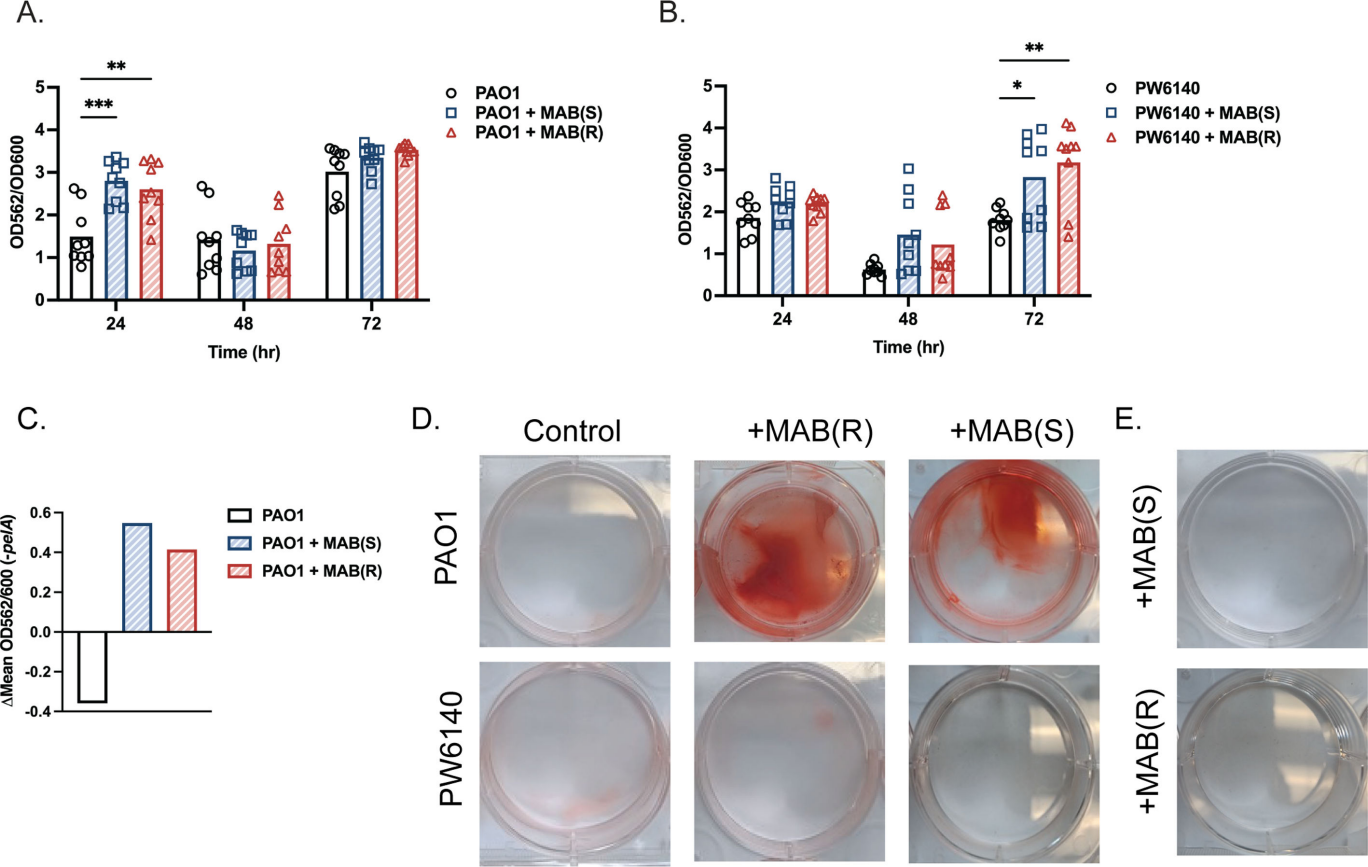
The Pel exopolysaccharide contributes to the increase in biofilm biomass during co-culture. *M. abscessus* ATCC 19977 smooth and rough morphotypes were co-cultured with *P. aeruginosa* strains PAO1 and PW6140 (PAO1*pelA::*ISphoAhah) in lysogeny broth (LB) medium in a 96-well plate for 24, 48, or 72 hours at 37°C. Biofilm biomass was then measured using crystal violet staining for (**A**) PAO1 or (**B**) PW6140 during single- and co-culture (*n* = 3 biological replicates and 3 technical replicates). Two-way ANOVA with Tukey’s multiple comparisons test was performed. (**C**) Mean difference in OD_562/600_ between PAO1 and PW6140 at the 24-hour time point. *M. abscessus* ATCC 19977 smooth and rough morphotypes were (**D**) co-cultured with *P. aeruginosa* strains PAO1 and PW6140 or (**E**) grown alone in LB medium in a 6-well plate for 24 hours before staining with Congo Red (1%). *P* < 0.05 (*), *P* < 0.01 (**), *P* < 0.001 (***).

The *P. aeruginosa* polysaccharide Pel has been shown to crosslink with eDNA (63). Due to the contribution of eDNA in *P. aeruginosa* and MAB dual biofilms, we hypothesized that Pel also plays a role in the promotion of *P. aeruginosa* in dual-species biofilms. We grew single- and dual-species biofilms with parent PAO1 or with a mutant of PAO1 with a transposon disruption in *pelA* (PW6140) and evaluated our established phenotype via the crystal violet assay. With the parent PAO1, we observed the expected significant increase in biomass at 24 hours with MAB(S) or MAB(R) (*P* = 0.0008, *P* = 0.0076) (Fig. 4A). With the disruption of Pel, this increase was lost at 24 hours, and the impact of the loss of Pel on biofilm formation was greater in dual-species biofilms than with *P. aeruginosa* alone (Fig. 4B and C). In support of these findings, Congo Red staining of Pel polysaccharide in 24-hour biofilms revealed that MAB stimulated the overproduction of Pel in dual-species cultures compared to single PAO1 cultures and that loss of *pelA* in the PAO1 background resulted in no Pel staining (Fig. 4D). Pel was not detected in the single biofilm MABS or MABR morphotype controls (Fig. 4E). Staining at earlier time points indicates that the increase in Pel in the dual-species biofilms begins as early as 6 hours of biofilm formation (Fig. S6B). These results were validated with a clean deletion mutant in *pelF* (Fig. S6B). Pel was also overproduced in the mucoid mPA08-31 strain when grown in dual biofilms with MAB (Fig. S6C).

Finally, we grew dual-species biofilms with MAB and either *P. aeruginosa* PA14 or FRD1. PA14 produces Pel but not Psl or alginate (64), which are two other major *P. aeruginosa* polysaccharides. We observed an increase in biofilm biomass with this strain during co-culture with MAB (Fig. S7A through C). With FRD1, a mucoid isolate that overproduces alginate (65), we observed only a slight increase in biomass at 24 hours of co-culture with MAB(R) (Fig. S7D through F). Taken together, these data indicate a particularly important role for Pel in early biofilm enhancement during co-culture with MAB.

### MAB promotes tolerance of *P. aeruginosa* to tobramycin

**Fig 5:**
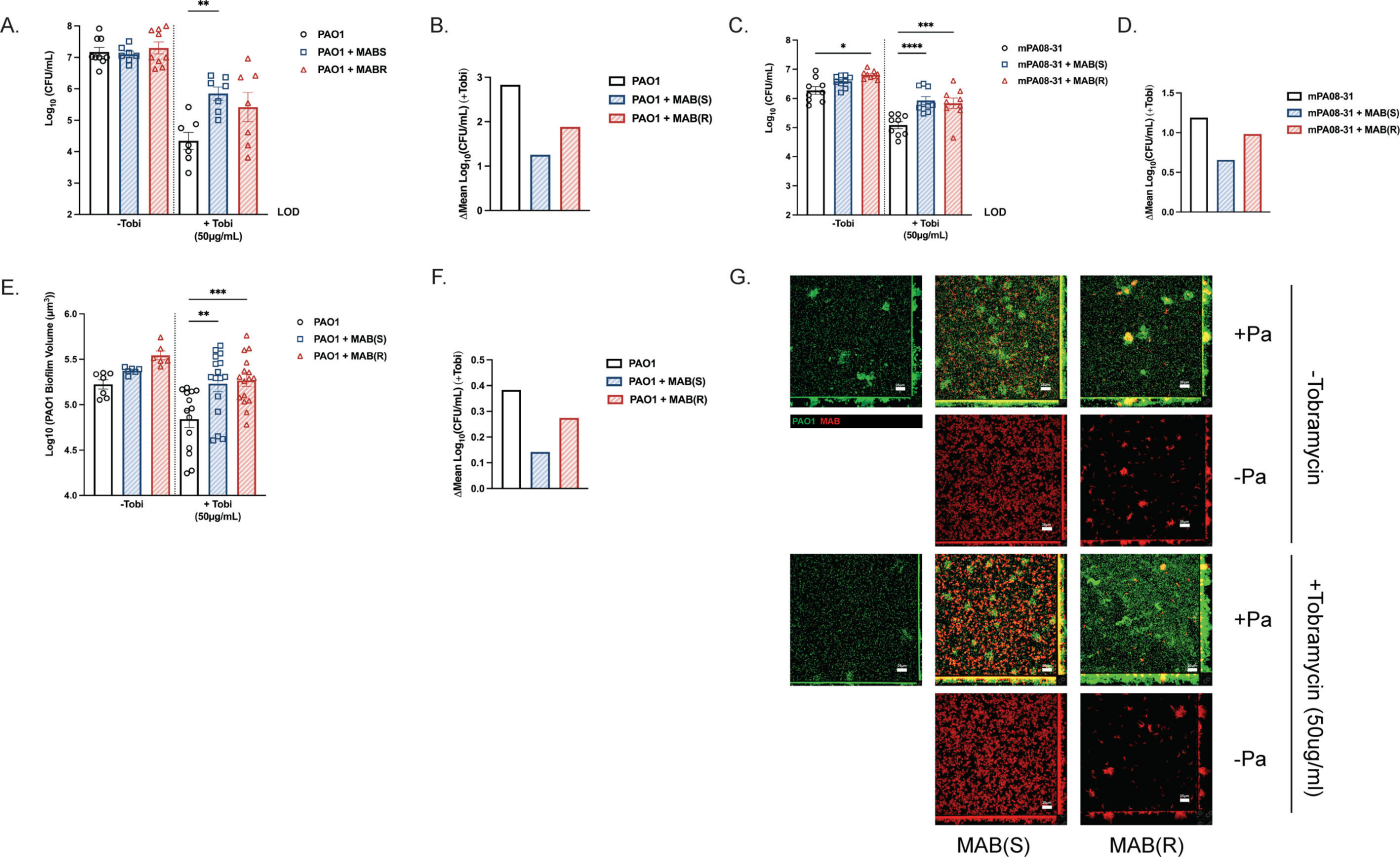
Co-culture of *P. aeruginosa* and MAB decreases the susceptibility of *P. aeruginosa* to tobramycin. *M. abscessus* ATCC 19977 smooth and rough morphotypes were co-cultured with PAO1 or mPA08-31 in lysogeny broth (LB) medium in a 96-well plate for 24 hours at 37°C. Biofilms were then treated for 1 hour with tobramycin (50 μg/mL) or 1× PBS. Viable colony-forming units of *P. aeruginosa* (**A**) PAO1 and (**C**) mPA08-31 during single- and dual-species culture with and without tobramycin treatment were measured via differential plating (*n* = 3 biological replicates and 3 technical replicates). One-way ANOVA with Tukey’s multiple comparisons test was performed. Mean differences in log_10_ CFU/mL with and without tobramycin treatment were plotted for (**B**) PAO1 and (**D**) mPA08-31. Fluorescently labeled *M. abscessus* ATCC 19977 smooth and rough morphotypes (mCherry+) were co-cultured with *P. aeruginosa* PAO1 (GFP+) in LB medium for 24 hours in an 8-well μSlide at 37°C (*n* = 2 biological replicates and 3 technical replicates). (**E**) Biofilm volume was quantified via BiofilmQ. One-way ANOVA with Šidák’s multiple comparisons test. (**F**) Mean difference in PAO1 biofilm volume with and without tobramycin treatment. (**G**) Structural composition of single- and dual-species biofilms with and without tobramycin treatment was evaluated via confocal imaging at 40× magnification. *P* < 0.05 (*), *P* < 0.01 (**), *P* < 0.001 (***).

The Pel exopolysaccharide is known to contribute to resistance to antimicrobials such as tobramycin (66–69). We therefore decided to test the susceptibility of *P. aeruginosa* to tobramycin treatment in a single-species biofilm compared to a dual-species biofilm with MAB. We grew single- and dual-species biofilms as in previous experiments and then replaced the medium with either phosphate-buffered saline (PBS) or tobramycin (50 µg/mL) for 1 hour. As expected from previous experiments, we observed no difference in *P. aeruginosa* CFUs with or without the presence of MAB. At the 24-hour time point, tobramycin treatment showed a decrease in live *P. aeruginosa* in single-species culture, but a lesser decrease in live *P. aeruginosa* in the presence of MAB. There was a significant difference between tobramycin-treated single-species culture and dual-species culture with MAB(S) for PAO1 (*P =* 0.0021), and both morphotypes for mPA08-31 (*P* ≤ 0.0001, *P* = 0.0003) (Fig. 5A through D). Changes in *P. aeruginosa* burden did not lead to any subsequent differences in the amount of MAB present in dual-species biofilms with PAO1 or mPA08-31 (Fig. S8A and C). To observe the effect of co-culture on the susceptibility of *P. aeruginosa* to tobramycin in terms of biofilm structure, we again performed confocal microscopy on single- and dual-species biofilms of *P. aeruginosa* PAO1 with or without a subsequent 1-hour tobramycin treatment (50 µg/mL). As expected from the CFU results, dual-species biofilms show significantly increased *P. aeruginosa* volume for both smooth and rough morphotypes (*P =* 0.0024, *P* = 0.0007) only after tobramycin treatment, with a smaller impact of treatment on dual-species biofilms than on single-species biofilms (Fig. 5E through G). These data indicate that in addition to thickening of the biofilm matrix and overproduction of Pel, co-culture with MAB also decreases the susceptibility of *P. aeruginosa* to the common anti-pseudomonal drug tobramycin.

### Presence of MAB modulates the expression of *P*. *aeruginosa* metabolism and biofilm-related genes

**Fig 6:**
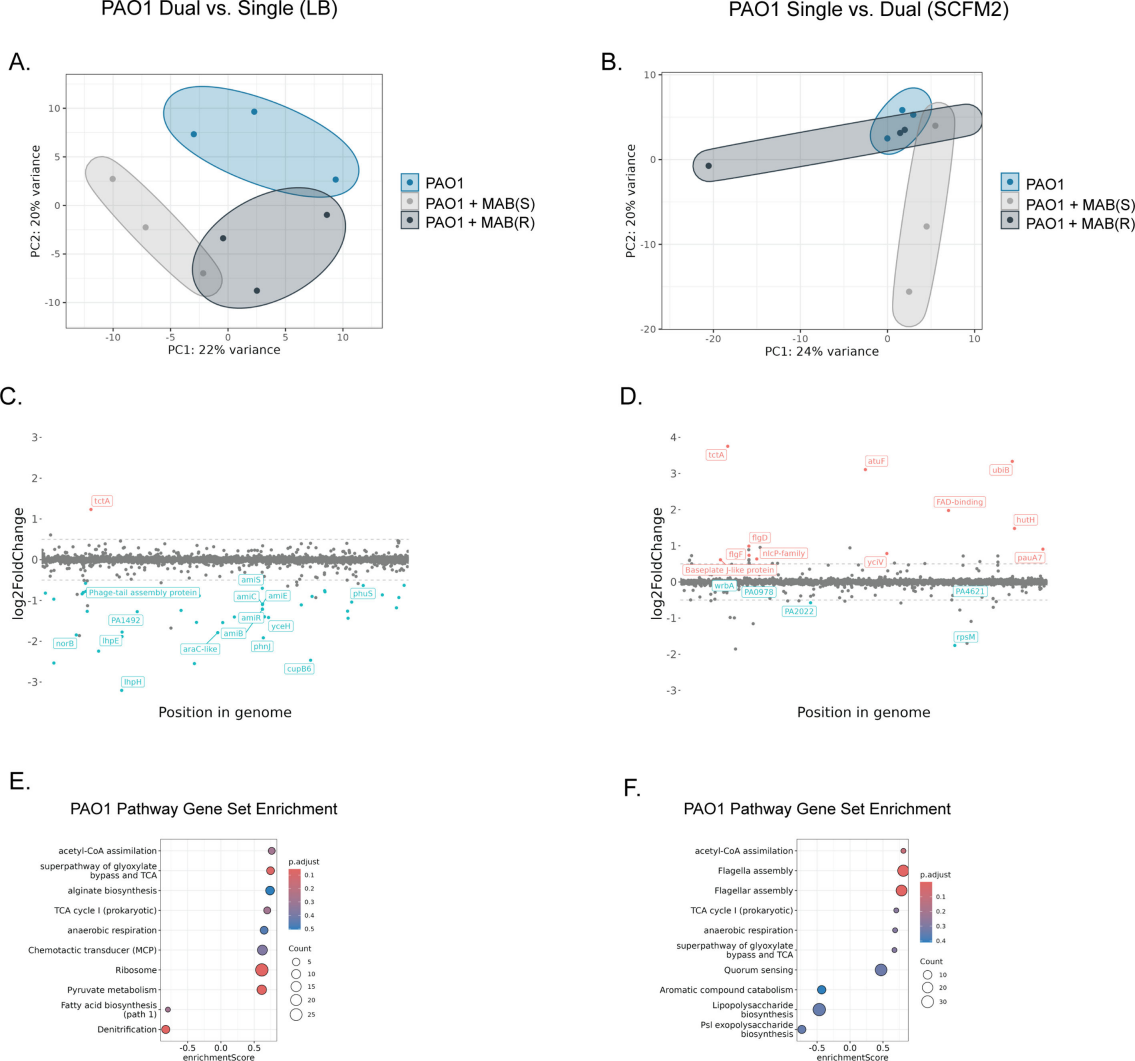
Co-culture of *P. aeruginosa* and MAB changes the transcriptomic profile of *P. aeruginosa. M. abscessus* ATCC 19977 smooth and rough morphotypes were co-cultured with *P. aeruginosa* PAO1 in LB medium in a 6-well plate for 48 hours at 37°C. Adherent cells were pooled (3 per sample) for RNA extraction and sequencing (*n* = 3). Principal component analysis of samples based on RNA sequencing data in (**A**) LB or (**B**) SCFM2, colored by infection group. *XY* graphs of differential expression significance in single or dual-species biofilms in (**C**) LB medium or (**D**) SCFM2, ordered by genome position, as determined via differential expression analysis using DESeq2. Red depicts genes significantly upregulated by *P. aeruginosa* during co-culture, while blue depicts significantly downregulated genes. Dotplots of gene set enrichment analysis using KEGG and Interpro signature databases for differential expression during co-culture in (**E**) LB or (**F**) SCFM2.

Our findings demonstrate that MAB alters the biofilm matrix of *P. aeruginosa* in a Pel- and eDNA-dependent manner. To probe the impact of MAB on the *P. aeruginosa* transcriptome, we performed RNA sequencing on *P. aeruginosa* biofilms grown either alone or in conjunction with MAB in both lysogeny broth (LB) and synthetic cystic fibrosis media (SCFM2). RNA harvested from biofilm samples was prepared for sequencing and sequenced at a read depth of ~20 million reads per sample. Principal component analysis of gene expression data indicated modest differences in the presence of MAB (Fig. 6A and B). Overall, differential expression analysis between samples showed that 57 genes are differentially regulated between dual species biofilms grown in LB, and 16 are differentially expressed when grown in SCFM2 compared to *P. aeruginosa* alone, with the top 15 represented in *xy* plots according to genome position (Fig. 6C and D). The most significantly upregulated gene shared between these growth conditions was PA0752 (*tctA*) (*P.adj.* = 2.38 × 10^−22^ in LB, *P.adj.* = 1.08 × 10^−24^ in SCFM2)*,* which encodes the transmembrane subunit of the TctABC tricarboxylate import system (70). This gene was also significantly upregulated in mPA08-31 grown in SCFM2 during co-culture (*P.adj.* = 9.40 × 10^−8^), although not when grown in LB (Fig. S10). In LB, 50 of the 57 differentially expressed genes were downregulated, including those of the aliphatic amidase operon (amiEBCRS), several genes of which are known to influence biofilm formation and other virulence phenotypes in *P. aeruginosa* (Fig. 6C). In SCFM2, we observed an increase in the expression of *flgF* and *flgD,* both of which encode for portions of the flagellar hook structure in *Pa,* known to influence biofilm development (71) (Fig. 6D). Gene set enrichment analysis for KEGG and Interpro signature pathways of PAO1 showed significant upregulation of pyruvate metabolism, ribosome, and superpathway of glyoxylate bypass and tricarboxylic acid (TCA) pathways, and downregulation of denitrification in LB (Fig. 6E). In SCFM2, only two pathways, both for flagellar assembly, were significantly upregulated. Psl and lipopolysaccharide (LPS) biosynthesis were also downregulated in this context (Fig. 6F). In both data sets, acetyl-CoA assimilation and TCA cycle-related pathways were enriched, although these did not reach statistical significance (Fig. 6E and F). Further investigations into the role of identified pathways during dual-species infections with MAB may help the development of antimicrobial strategies for polymicrobial infections and aid in our understanding of the increase in infection severity seen in patients when both organisms are present.

## DISCUSSION

In this study, we sought to explore interactions between *P. aeruginosa* and MAB to better understand how they might contribute to the severity of CF infection. We established a dual-species biofilm model where we measured biofilm biomass, architecture, and tolerance to antimicrobial treatment. Taken together, our results indicate that despite rapid killing of MAB during co-culture, overall biofilm biomass increased for a variety of *P. aeruginosa* strains used and was independent of MAB morphotype. This increase in biomass was not the result of a corresponding increase in bacterial cell number; therefore, we investigated the contribution of biofilm matrix components. We found that DNase treatment abrogated the dual-species increase in biomass at early time points, indicating that eDNA is likely involved. Loss of phenotype after disruption of a major exopolysaccharide of non-mucoid *P. aeruginosa* strains, Pel, and overproduction of this polysaccharide in dual biofilms indicated that Pel is critical for *P. aeruginosa* promotion in early biofilm development with MAB. The DNase treatment results mirrored those of the Pel disruption, suggesting that eDNA and Pel are collectively acting to increase dual-species biofilm biomass at early time points. Co-culture of these two organisms also increased the tolerance of *P. aeruginosa* to tobramycin, an anti-pseudomonal antibiotic commonly used in a CF context. Finally, we performed transcriptomic analysis and found that in the presence of MAB, *P. aeruginosa* differentially expresses genes involved in central metabolism and known to impact biofilm formation in both LB and artificial CF sputum medium. Ultimately, these results indicate that dual-species interactions between *P. aeruginosa* and MAB may contribute to persistence during pulmonary infection and to the recalcitrance of infections even with appropriate antibiotic treatment.

Initially, we found that co-culture of both lab-adapted and clinical strains of *P. aeruginosa* with either the rough or smooth morphotypes of MAB resulted in a thicker biofilm than either of these species alone. Consistent with previous reports of *P. aeruginosa* and MAB co-culture, imaging of biofilm structure showed MAB primarily on the substratum, with *P. aeruginosa* localized throughout the biofilm (58). This might indicate that MAB is protected by the *P. aeruginosa* matrix from either antimicrobial treatment, oxidative stress, or phagocytosis, although we did not measure these directly. Cooperative dual-species interactions with an increase in total biomass are often the result of promotion of growth for one or both species involved. However, we found that there was no significant increase in the growth of *P. aeruginosa* and a dramatic decrease in live MAB present during co-culture. This indicated that it was likely an extracellular matrix component that was responsible for the increase in biofilm formation. Because of the relative proportion of MAB compared to *P. aeruginosa* in the biofilms at later time points and the increase in biomass regardless of the MAB morphotype, we felt that the matrix component was likely either being produced by *P. aeruginosa* or was a result of the killing of MAB (such as eDNA). In line with this, DNase degradation experiments indicated that eDNA likely plays a role in our dual-species phenotype, although it did not completely abrogate the increase in biofilm formation.

Because DNase treatment contributed to a partial reduction in our dual-species phenotype, we also wanted to determine if Pel polysaccharide, which crosslinks with eDNA, was involved in enhanced *P. aeruginosa* biofilm development (63). Disruption of Pel abolished the increase in biofilm when MAB was present at the 24-hour time point, and Pel was overproduced in dual cultures, indicating that Pel is likely primarily responsible for this increase, particularly at earlier time points. We also observed that PA14, which produces Pel but lacks the locus for the other major exopolysaccharide, Psl, showed an increase in biofilm production in the presence of MAB. FRD1, an alginate overproducer known to express smaller amounts of Psl and Pel, showed the least amount of increase in biofilm formation, consistent with our proposed mechanism of a Pel-dependent increase (65).

Our results indicate that both Pel and eDNA are involved in the dual-species increase in biofilm biomass, particularly at earlier time points. Direct ionic binding between eDNA and Pel has been demonstrated, and interactions between these molecules are thought to directly contribute to *P. aeruginosa* biofilm structure and rheology (63, 67, 72). Although we did not directly investigate any changes in the structural interactions between these two molecules in the dual-species biofilm of MAB and *P. aeruginosa,* it is possible that the biofilm structure is being strengthened by interactions between the Pel derived from *P. aeruginosa* and the eDNA from MAB, providing a mechanism by which both species might show increased persistence in a host environment despite direct antagonism. This is also consistent with reports that these two organisms do not correlate with initial acquisition but do result in more severe and recalcitrant infections (51–53).

Both Pel and eDNA, individually and when bound, are known to contribute to increased tolerance of *P. aeruginosa* biofilms to aminoglycosides, including the CF-relevant antibiotic tobramycin (67–69, 73). We therefore hypothesized that this is the mechanism by which co-culture with MAB is increasing tolerance to tobramycin in our dual-species biofilms. However, this could also be mediated by a variety of other mechanisms, including changes in metabolism or increased expression of efflux pumps. We did not observe an increase in these systems in the RNA-sequencing analysis; however, this analysis was performed at a later time point than the tobramycin assays, and these changes may have been captured by studying transcriptomic changes at a variety of time points. As this is an important topic for patient outcomes, particularly for pwCF for whom inhaled tobramycin is a part of standard treatment regimens, further investigation into the basis for this increase in antibiotic tolerance would be warranted.

In transcriptomic experiments, we found that *tctA,* the gene that encodes the transmembrane component of a tripartite tricarboxylate import system, is significantly upregulated by *P. aeruginosa* in dual-species biofilms. TctABC is used in the uptake of citrate and *cis*-aconitase in *P. aeruginosa*. TctD and TctE, regulatory elements of the same system, have been implicated in aminoglycoside tolerance and biofilm formation in *Pseudomonas aeruginosa* PA14 in the presence of citrate (74). Furthermore, citrate-mediated cross-feeding with impacts on virulence has also been demonstrated between *lasR*− and *lasR*+ strains of *P. aeruginosa* strains, as well as between *P. aeruginosa* and *Staphylococcus aureus* (75).

Gene set enrichment analysis showed significant upregulation of pyruvate metabolism and the superpathway of glyoxylate bypass and TCA, a pathway that was also enriched in SCFM2, although not to a significant degree. Pyruvate is required for the formation of biofilms in *P. aeruginosa* and coordinates the metabolic flux between the TCA cycle and glyoxylate shunt in this organism (76, 77). Our observed increase in *tctA* expression, together with the upregulation of pyruvate metabolism and tricarboxylic acid cycle pathways, suggests that the presence of MAB causes shifts in central metabolic processes in *P. aeruginosa* and may indicate that *P. aeruginosa* is scavenging nutrients from MAB, either via secreted metabolites or following bacterial cell death, as a means of increasing biofilm formation.

In our transcriptomic analysis after growth in LB, we observed a nearly uniform decrease in the *amiEBCRS* operon of *P. aeruginosa* during co-culture with MAB. The small non-coding RNA *amiL* encoded on this operon is negatively regulated by both *las* and *rhl* systems and is known to regulate a wide variety of virulence phenotypes. Deletion of AmiL increases elastase activity, pyocyanin production, and relative biofilm production (78). AmiE overexpression in PA14 causes a decrease in biofilm thickness and twitching motility and a likewise decrease in molecules involved in virulence, including HCN and pyocyanin (79). Finally, AmiC is responsible for biofilm dispersal after exposure to human C-type natriuretic peptide, again indicating an inverse relationship to biofilm formation in *P. aeruginosa* (80).

Finally, in the SCFM2 condition, we observed upregulation of flagellar biosynthesis pathways and the downregulation of Psl and LPS biosynthesis. Two genes significantly upregulated in the flagellar biosynthesis pathway were *flgD* and *flgF,* both of which are part of the flagellar hook structure. FlgD has been implicated in biofilm development and maturation after the initial adherence step, and deletion of *flgE,* a gene of the same system, results in decreased production of biofilm-related polysaccharides, including Pel (71). Because Psl, Pel, and LPS share common biosynthetic precursors, the production of these polysaccharides is often inversely related, with induction of Pel reducing Psl and LPS synthesis. While this phenomenon is generally not transcriptionally controlled, the downregulation of Psl and LPS biosynthesis pathways during dual-species co-culture further supports that Pel is the major exopolysaccharide mediating this interaction (81).

Although this study included several implications for further research and treatment options, it was limited by its restriction to an *in vitro* biofilm model in a rich medium. Although several of the transcriptomic changes were shared between rich medium and synthetic CF sputum medium, further studies in more relevant conditions for CF disease, including a cell culture model, are warranted to confirm that similar interactions are occurring in clinical contexts.

In summary, we have demonstrated that co-culture of two relevant late-stage CF pathogens, *P. aeruginosa* and MAB, contributes to disease-relevant phenotypes, including an increase in biofilm formation and antimicrobial tolerance. We have also demonstrated that eDNA and Pel, two components of the biofilm matrix known to contribute to infection recalcitrance, are involved in the increase in biofilm formation during dual-species infection. Further investigations into the impact of MAB on central metabolism and molecular determinants of *P. aeruginosa* biofilm development are needed to identify vulnerable pathways that can be exploited in the treatment of these complicated infections.

## MATERIALS AND METHODS

### Strains and growth conditions

*P. aeruginosa* strains used in this study include PAO1 (provided by D. Wozniak, The Ohio State University) and PA14, two widely used non-mucoid model strains, mPA08-31, a mucoid clinical isolate (provided by S. Birket, University of Alabama at Birmingham), and FRD1, a model mucoid strain. *P. aeruginosa* mPA08-31^GFP+^ and PAO1^GFP+^ were constructed by transforming parent strains with plasmid pSMC2 (provided by G. O’Toole, Dartmouth College). Strain PW6140 (PAO1*pelA::*ISphoAhah) was obtained from the Manoil Lab PA two-allele mutant library (82). Strain PAO1Δ*pelF* is a clean deletion mutant (provided by E. Gloag, Virginia Tech) (83). *P. aeruginosa* strains were maintained on *Pseudomonas* isolation agar (20 g peptone, 1.4 g magnesium chloride, 10 g potassium sulfate, 25 mg Irgasan, 21.1 g agar, and 12.5 g LB per liter), then cultured in lysogeny broth (10 g tryptone, 5 g yeast extract, and 10 g sodium chloride per liter), and incubated at 37°C with shaking at 250 rpm.

*M. abscessus* strains used in this study include ATCC 19977 rough and smooth isotypes, obtained from the American Type Culture Collection. MAB(S)^mCherry+^ and MAB(R)^mCherry+^ were constructed by transforming parent strains with plasmid pMSP12::mCherry, obtained from Addgene (#30169), using previously described methods (84). *M. abscessus* strains were maintained on 7H10 agar (Remel) with 10% oleic acid-albumin-dextrose-catalase (OADC) supplement (BD) and glycerol, then cultured in 7H9 medium (Remel) with 0.05% Tween, 10% albumin-dextrose-catalase supplement (BD), and glycerol, and incubated at 37°C with shaking at 80 rpm.

### Static biofilm formation assays

*In vitro* biofilm assays were performed according to an established microtiter assay protocol with some modifications (85). Overnight cultures of *P. aeruginosa* were subcultured 1:100 in LB and grown to mid-log phase (OD at 600 nm = 0.4–0.6). Broth stocks of MAB grown to stationary phase in 7H9 (+Tween, OADC) were subcultured 1:100 into fresh medium and grown overnight (37°C at 80 rpm) to mid-log phase (OD at 600 nm = 0.4–0.6). MAB was pelleted (5,000*g* for 5 min) and resuspended in LB. Both species were inoculated either separately or together into a flat-bottom 96-well microtiter dish (Corning) containing LB at a final density of ~10^6^ CFU/mL for *P. aeruginosa* and ~10^7^ CFU/mL for MAB to account for differences in growth rate (200 μL/well). Biofilms were incubated without shaking at 37°C for the time specified for each assay.

To quantify the resulting biofilm, plates were washed, stained with 0.1% crystal violet, and dissolved in 30% acetic acid. Absorbance was first measured at 600 nm prior to washing and then at 562 nm after crystal violet staining to quantify biofilm biomass using the Synergy HTX Multi-Mode Microplate Reader (BioTek). Biofilm formation is expressed as biomass normalized to growth (OD_562_/OD_600_) unless otherwise specified. To stain the Pel polysaccharide, 4-, 6-, and 16-hour biofilms were grown in 6-well dishes, gently washed twice with PBS (3 mL), and dried for 15 min. Next, Congo Red (0.1% Congo Red in 50% ethanol, 3 mL) was added to dried biofilms for 15 min, followed by two PBS washes. Biofilms were allowed to dry prior to imaging.

### Quantification of *P. aeruginosa* and MAB during co-culture

Biofilms were prepared as described above in 96-well microtiter dishes. To quantify colony-forming units of each species, adherent biofilm cells were washed twice with phosphate-buffered saline, scraped, and resuspended in 200 µL of PBS before serial dilution and selective plating using the track dilution method (86), with a limit of detection of 10^2^. A selective plating scheme of MacConkey agar (Remel) to grow *P. aeruginosa* alone and Columbia CNA agar (BD) with 5% sheep’s blood (Hemostat Laboratories) to grow MAB was used as previously described (57, 58)

### Confocal microscopy

Single- and dual-species biofilms were prepared as described above in a sterile 8-well treated μSlide (Ibidi) using fluorescently labeled strains *P. aeruginosa* PAO1^GFP+^, mPA08-31^GFP+^, MAB(S)^mCherry+^, and MAB(R)^mCherry+^. Adherent biofilm cells were then washed twice with phosphate-buffered saline and fixed using Fluoromount-G (Invitrogen). Confocal laser scanning microscopy was performed using a Nikon-A1R HD25 confocal laser microscope (Nikon) at 40× magnification. Images were acquired and processed using the NIS-elements 5.0 software. For analysis, images were first denoised using the Denoise.ai feature of NIS Elements software. Image stacks were then imported into a biofilm quantification software, BiofilmQ (62), as ND2 files to preserve metadata. Image stacks were thresholded for each channel individually, differentiating between biofilm and background signal. To analyze the properties of each biofilm volume with spatial resolution, BiofilmQ employs a cube-based method to define localization. Defined volumes from fluorescent images were dissected into individual pseudo-cell cubes with a side length of 3 voxels (0.94 µm), for which parameter calculations were then performed. Metrics calculated included mean biofilm volume (µm^3^/area), mean biofilm height (µm/area), and distance to substrate (µm/voxel) for each 1,024 × 1,024 pixel (322 × 322 µm) substrate area, with the substrate being the bottom surface of the 8-well slide. Three images were analyzed per biofilm for at least two biological replicates.

### DNase treatment

For the quantification of biofilm biomass with and without DNase treatment, single- and dual-species biofilms were grown as detailed above for 24, 48, or 72 hours. OD at 600 nm was taken before the supernatant was removed and replaced with DNase (25 U/mL) in PBS. Biofilms were incubated with DNase at 37°C for 1 hour before performing a crystal violet assay as previously described. For confocal imaging, single- and dual-species biofilms were grown for 24 hours for PAO1^GFP+^ biofilms and 72 hours for mPA08-31^GFP+^ biofilms. Supernatant was removed and replaced with DNase (25 U/mL) in PBS. Adherent biofilm cells were then washed twice with PBS and fixed using Fluoromount-G (Invitrogen) before performing imaging and quantification as described above.

### Tobramycin susceptibility

For the quantification of biofilm biomass with and without tobramycin treatment, single- and dual-species biofilms were grown as detailed above for 24 hours. The supernatant was then removed and replaced with tobramycin (50 µg/mL) in PBS or a PBS control. Biofilms were incubated with tobramycin treatment at 37°C for 1 hour before performing a crystal violet assay as previously described. For confocal imaging, single- and dual-species biofilms were grown for 24 hours. The supernatant was removed and replaced with tobramycin (50 µg/mL) in PBS. Adherent biofilm cells were then washed twice with phosphate-buffered saline and fixed using Fluoromount-G (Invitrogen) before performing imaging and quantification as described above.

### RNA sequencing and analysis

For single- and dual-species RNA sequencing, biofilms were prepared as described above with some modifications. Overnight cultures of *P. aeruginosa* were subcultured 1:100 in LB and grown to mid-log phase (OD at 600 nm = 0.4–0.6). Broth stocks of MAB grown to stationary phase in 7H9 (+Tween, OADC) were subcultured 1:100 in fresh medium and grown overnight (37°C at 80 rpm) to mid-log phase (OD at 600 nm = 0.4–0.6). MAB was pelleted (5,000*g* for 5 min) and resuspended in LB. Both species were inoculated either separately or together into a 6-well microtiter dish (Corning) containing LB at final densities of ~10^6^ CFU/mL for *P. aeruginosa* and ~10^7^ CFU/mL for MAB (3 mL/well). Biofilms were incubated without shaking at 37°C for 48 hours before being washed twice with PBS. Adherent cells from three wells were pooled for each sample. Samples were pelleted and resuspended in TRIzol reagent (Invitrogen) before bead beating (0.1 mm silica) to lyse cells. RNA was extracted according to a standard protocol (87).

Isolated RNA was sent to SeqCenter (Pittsburgh, PA, USA) for rRNA and tRNA depletion, sequencing, and analysis. Reads were aligned to either the PAO1 or mPA08-31 genome as appropriate using HISAT2 (v. 2.2.0). Read counts were obtained via Subread featureCounts (v.2.0.1), and differential expression analysis was performed with DESeq2 with an adjusted *P*-value cutoff of <0.05. Gene set enrichment analysis was performed using clusterProfiler (88) (v. 3.20) with *Pseudomonas aeruginosa* PAO1 (89). The Pathway Annotations list was obtained from pseudomonas.com. This combines KEGG and InterPro signature annotations from Pseudocyc, Metacyc, and KEGG databases. Dotplots were generated using enrichplot (90) (v. 3.21).

### Statistical analyses

Unless otherwise noted, graphs represent sample means ± standard error of the mean. For nonparametric analyses, differences between groups were analyzed by the Kruskal-Wallis test with the uncorrected Dunn’s test for multiple comparisons. For normally distributed data sets (as determined by the Shapiro-Wilk normality test), a one-way analysis of variance was used with Tukey’s multiple comparison test. Those experiments with two factors (for example, CFU changes over time) were analyzed via two-way ANOVA. Outliers were detected via the ROUT method (Q, 1%) and excluded from the analysis. For all relevant statistical analyses, an alpha value of 0.5 was used, and significance was denoted as follows: *P < 0.05, **P < 0.01, ***P < 0.001, and ****P < 0.0001. For viable colony counts, data were transformed (*Y* = log_10_[*Y*]) to achieve normally distributed data appropriate for use with a two-way ANOVA. All statistical tests were performed using GraphPad Prism 10 (San Diego, CA, USA).

## Data Availability

The raw sequencing data from the current study are available in the Sequence Read Archive (SRA) under the BioProject PRJNA1073745.

## SUPPLEMENTAL MATERIAL

Data S1 msystems.00270-26-s0001.csv Gene counts mapped to *P. aeruginosa* PAO1 in RNA-sequencing experiments.

Data S2 msystems.00270-26-s0002.csv Gene counts mapped to *P. aeruginosa* mPA08-31 in RNA-sequencing experiments.

Data S3 msystems.00270-26-s0003.csv Differential expression of genes from *P. aeruginosa* PAO1 in single- and dual-species biofilms in LB.

Data S4 msystems.00270-26-s0004.csv Differential expression of genes from *P. aeruginosa* PAO1 in single- and dual-species biofilms in SCFM2.

Data S5 msystems.00270-26-s0005.csv Differential expression of genes from *P. aeruginosa* mPA08-31 in single- and dual-species biofilms in LB.

Data S6 msystems.00270-26-s0006.csv Differential expression of genes from *P. aeruginosa* mPA08-31 in single- and dual-species biofilms in SCFM2.

Data S7 msystems.00270-26-s0007.xlsx Mean differences of confocal images.

Supplemental Material msystems.00270-26-s0008.pdf Supplemental figures and legends for Data S1 to S7.

ASM does not own the copyrights to Supplemental Material that may be linked to, or accessed through, an article. The authors have granted ASM a non-exclusive, world-wide license to publish the Supplemental Material files. Please contact the corresponding author directly for reuse.
