# Supplementary material for: The Pel polysaccharide modulates interspecies interactions between Pseudomonas aeruginosa and Mycobacterium abscessus
Source: mSystems. 2026 May 18;11(6):e00270-26. doi: 10.1128/msystems.00270-26 (PMC13289088; doi:10.1128/msystems.00270-26)
Supplement: Supplemental Material — Supplemental figures and legends for Data S1 to S7. [file msystems.00270-26-s0008.pdf]

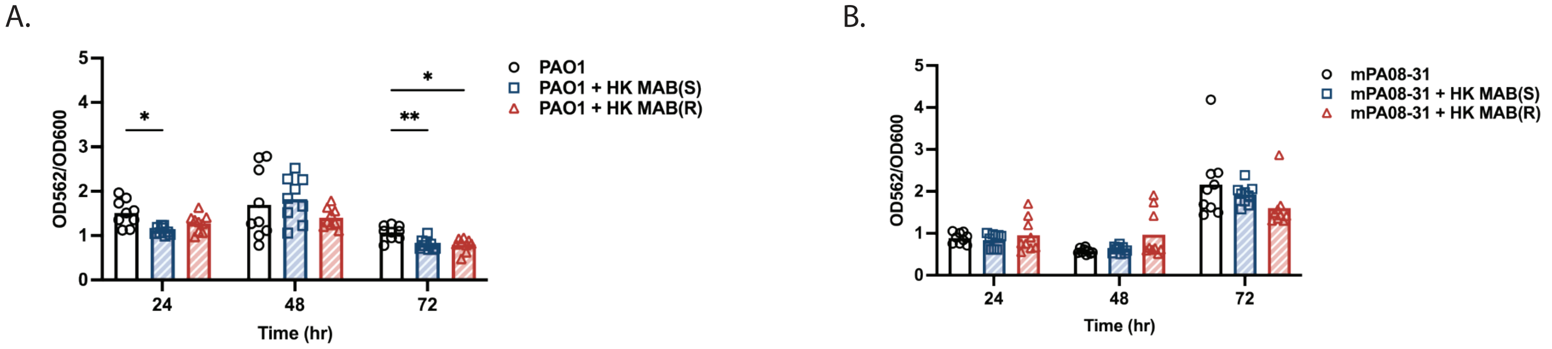

Supplementary Figure 1. Heat-killed MAB does not increase biofilm formation.
M. abscessus ATCC 19977 smooth and rough morphotypes were heat-killed at 80°C for 30 minutes before addition to either of two P. aeruginosa strains (PAO1 or mPA08-31) in LB medium in a 96-well plate for 24, 48, or 72 hours at 37 °C. Biofilm biomass was then measured using crystal violet staining (n = 3 biological replicates, 3 technical). Two-way ANOVA with Tukey's multiple comparisons test.

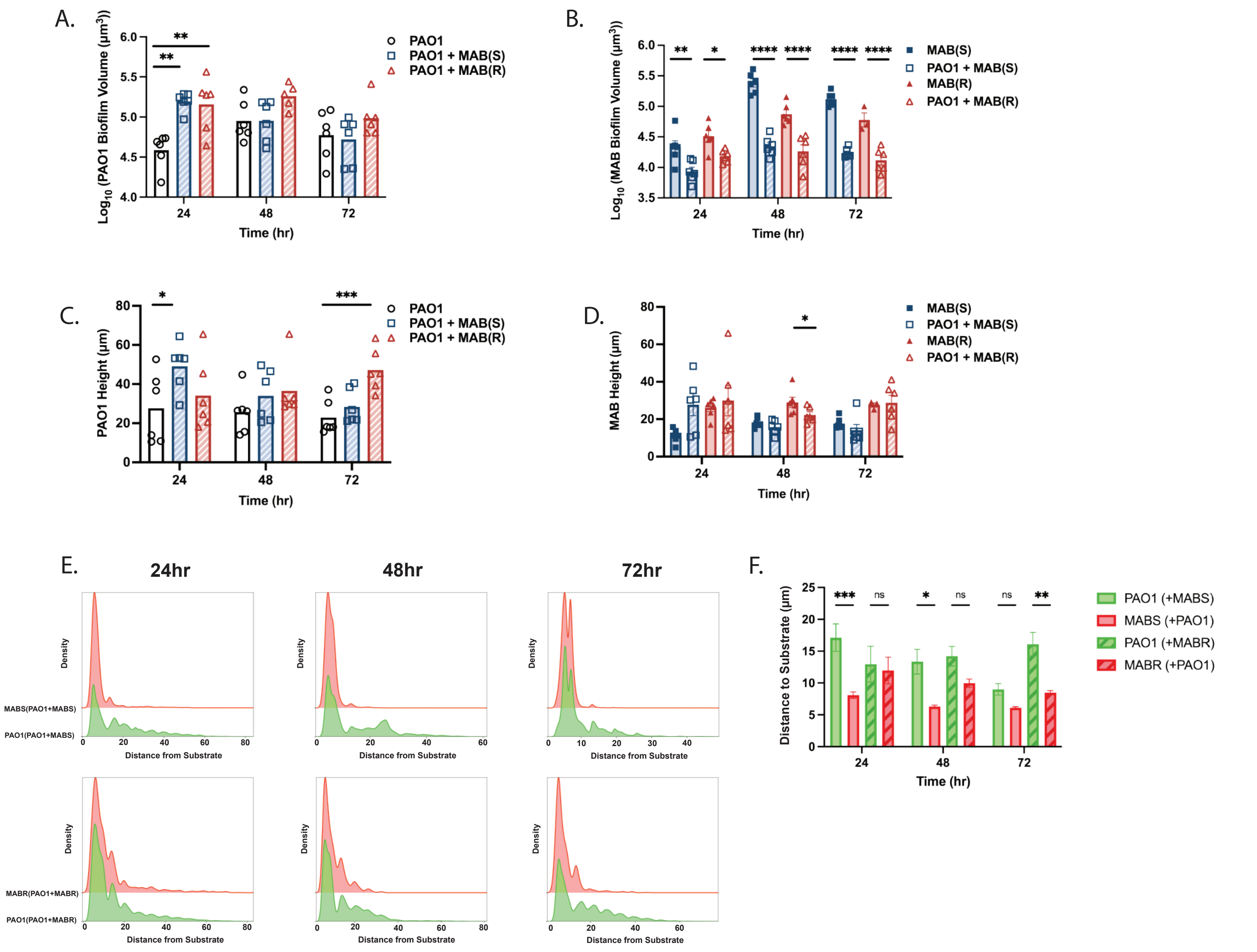

Supplementary Figure 2. Imaging of dual species *P. aeruginosa* PAO1 and MAB biofilms
Fluorescently labeled *M. abscessus* ATCC19977 smooth and rough morphotypes (mCherry+) were co-cultured with *P. aeruginosa* PAO1 (GFP+) in LB medium in an 8-well µSlide for 24, 48, or 72 hours at 37 °C (n = 2 biological replicates, 3 technical). A) Volume and B) height of *P. aeruginosa* PAO1 and C) volume and D) height of *M. abscessus* in single- and dual-species biofilms were quantified via BiofilmQ. One-way ANOVA with Tukey's multiple comparisons test. E) Histograms of pixel distribution of *P. aeruginosa* PAO1 and *M. abscessus* distance to substrate at each timepoint for rough and smooth morphotypes. F) Mean distance to substrate summarized. (n = 2 biological replicates, 3 technical).

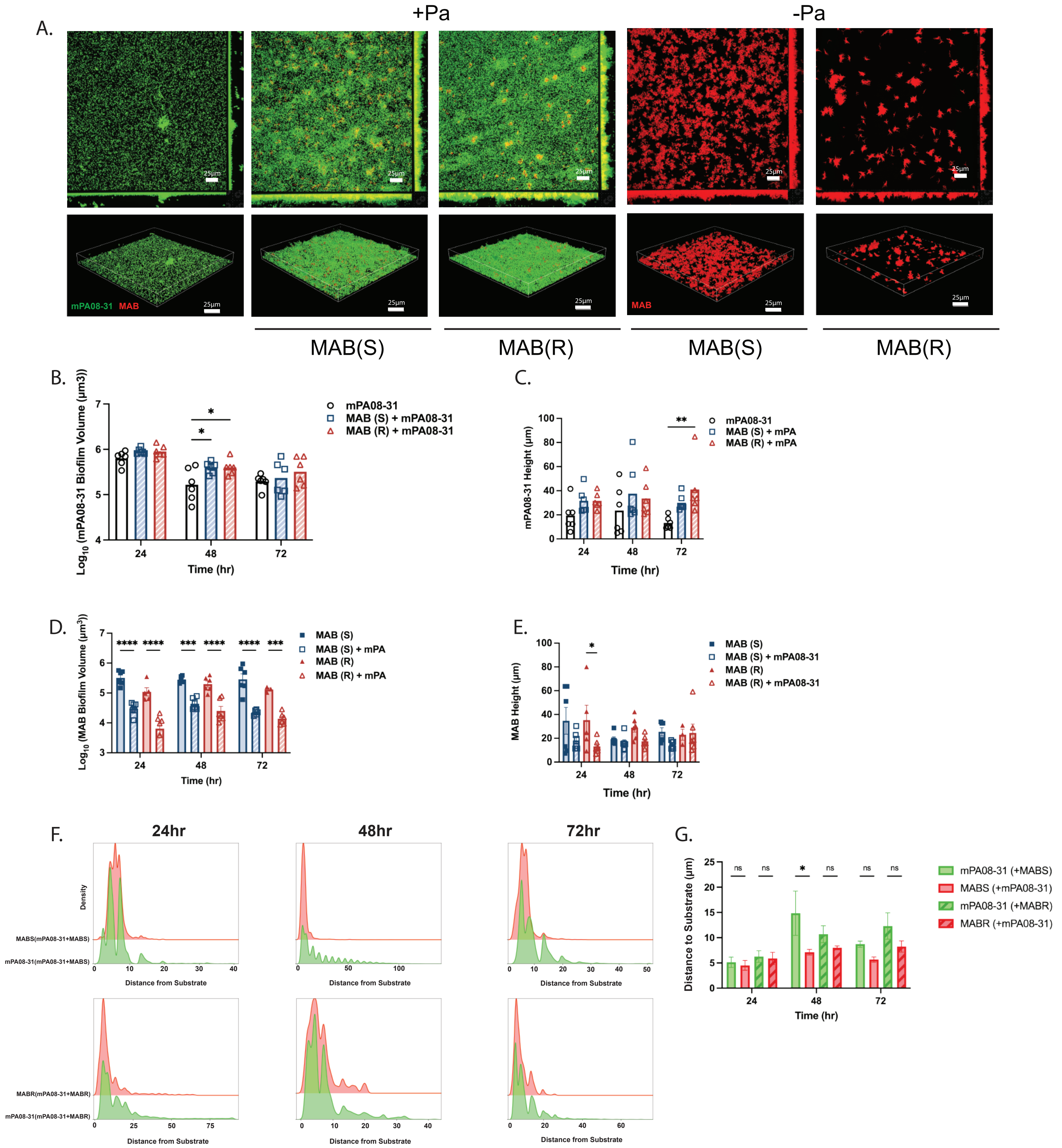

Supplementary Figure 3. Imaging of dual species *P. aeruginosa* mPA08-31 and MAB biofilms
Fluorescently labeled *M. abscessus* ATCC19977 smooth and rough morphotypes (mCherry+) were co-cultured with *P. aeruginosa* mPA08-31 (GFP+) in LB medium in an 8-well µSlide for 24 hours at 37 °C (n = 2 biological replicates, 3 technical). A) Structural composition of single- and dual-species biofilms was evaluated via confocal imaging at 40X magnification. B) Volume and C) height of *P. aeruginosa* mPA08-31 and D) volume and E) height of *M. abscessus* in single- and dual-species biofilms were quantified via BiofilmQ. One-way ANOVA with Tukey's multiple comparisons test. F) Histograms of pixel distribution of *P. aeruginosa* PAO1 and *M. abscessus* distance to substrate at each timepoint for rough and smooth morphotypes. G) Mean distance to substrate summarized. (n = 2 biological replicates, 3 technical).

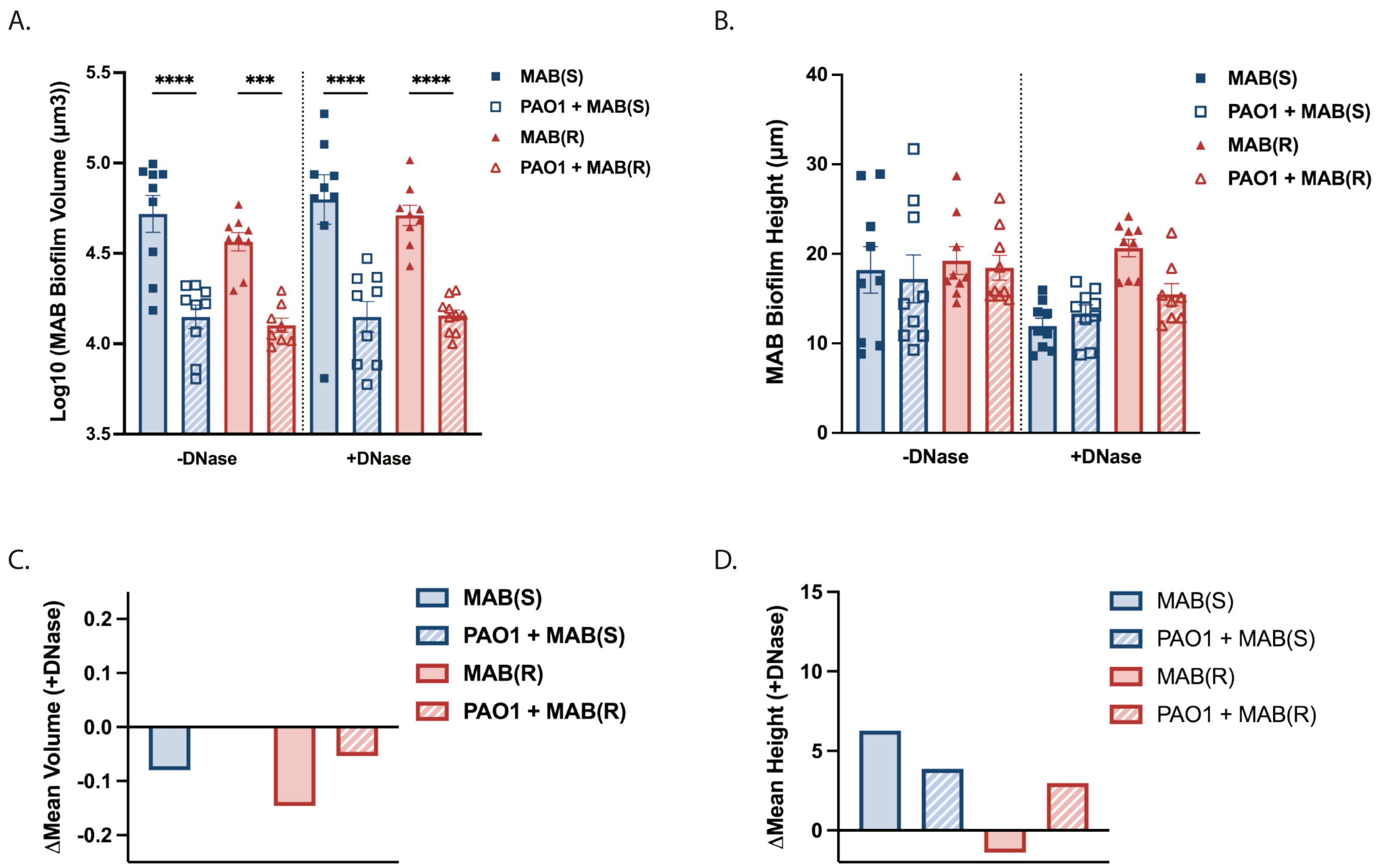

Supplementary Figure 4. DNase treatment partially abrogates dual-species cooperativity between *P. aeruginosa* PAO1 and MAB.

M. abscessus ATCC 19977 smooth and rough morphotypes were co-cultured with *P. aeruginosa* strain PAO1 in LB medium in a 96-well plate for 24, 48, or 72 hours at 37 °C. Biofilms were then treated for 1 hour with DNase (25U/mL) or PBS. Structural composition of single- and dual-species biofilms with and without DNase treatment was evaluated via confocal imaging at 40X magnification. A,B) Volume and height of *P. aeruginosa* PAO1 and A,B) *M. abscessus* single- and dual-species biofilms were quantified via BiofilmQ. (n = 3 biological replicates, 3 technical). Kruskal-Wallis with Dunn's multiple comparisons test. Difference in c) mean volume and D) mean height between non-DNase treated and DNase treated samples.

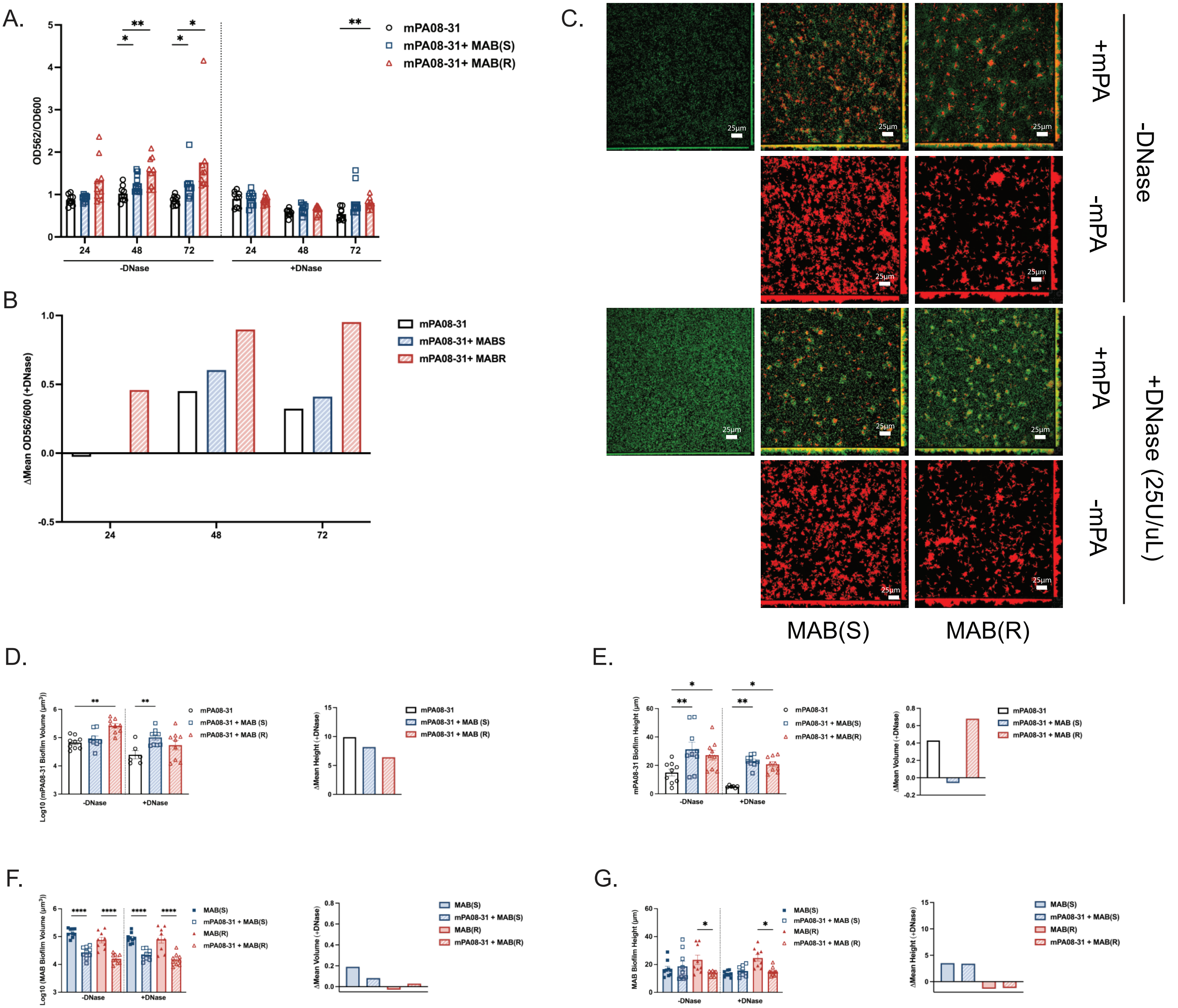

Supplementary Figure 5. DNase treatment partially abrogates dual-species cooperativity between *P. aeruginosa* mPA0831 and MAB.

M. abscessus ATCC 19977 smooth and rough morphotypes were co-cultured with *P. aeruginosa* strain mPA08-31 in LB medium in a 96-well plate for 24, 48, or 72 hours at 37 °C. Biofilms were then treated for 1 hour with DNase (25U/mL) or PBS. A) Biofilm biomass was then measured using crystal violet staining during single- and co-culture (n = 3 biological replicates, 3 technical). Two-way ANOVA with Tukey's multiple comparisons test. B) Mean difference in OD562/OD600 between DNase and non-DNase treated biofilms. Fluorescently labeled *M. abscessus* ATCC19977 smooth and rough morphotypes (mCherry+) were co-cultured with *P. aeruginosa* mPA08-31 GFP+ for 72 hours in an 8-well µSlide at 37 °C (n = 2 biological replicates, 3 technical). C) Structural composition of single- and dual-species biofilms with and without DNase treatment was evaluated via confocal imaging at 40X magnification. D,E) Volume and height (with mean differences between treatment and no treatment) of *P. aeruginosa* mPA08-31 and F,G) *M. abscessus* single- and dual-species biofilms were quantified via BiofilmQ (n = 3 biological replicates, 3 technical). Kruskal-Wallis with Dunn's multiple comparisons test.

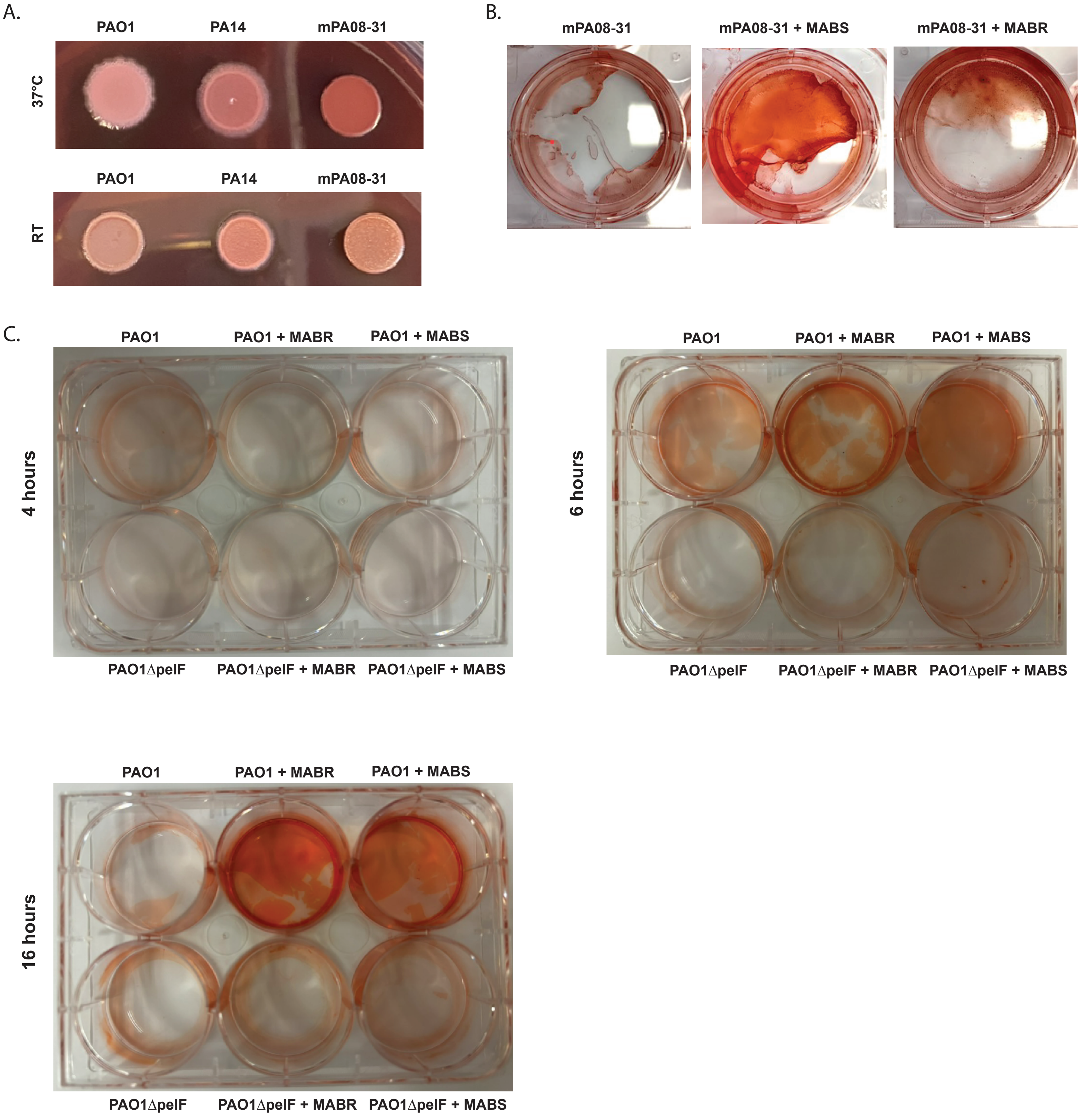

Supplementary Figure 6. ***P. aeruginosa*** **morphology on Congo Red plates**
A)*P. aeruginosa* strains PAO1, PA14, and mPA08-31 were grown on Congo red plates (LB, no NaCl, 40µg/mL Congo red and 20µg/mL Coomassie blue) at either 37 °C or room temperature for 48 hours. B) *P. aeruginosa* strain mPA08-31 was grown alone or with *M. abscessus* for 72 hours in 6 well plates before staining with Congo Red (1% in 50% ethanol and 50% water). C) *P. aeruginosa* strains PAO1 and PAO1Δ*pelF* were grown with or without *M. abscessus* smooth and rough morphotypes for 4, 6, or 16 hours before staining with Congo Red (1% in 50% ethanol and water).

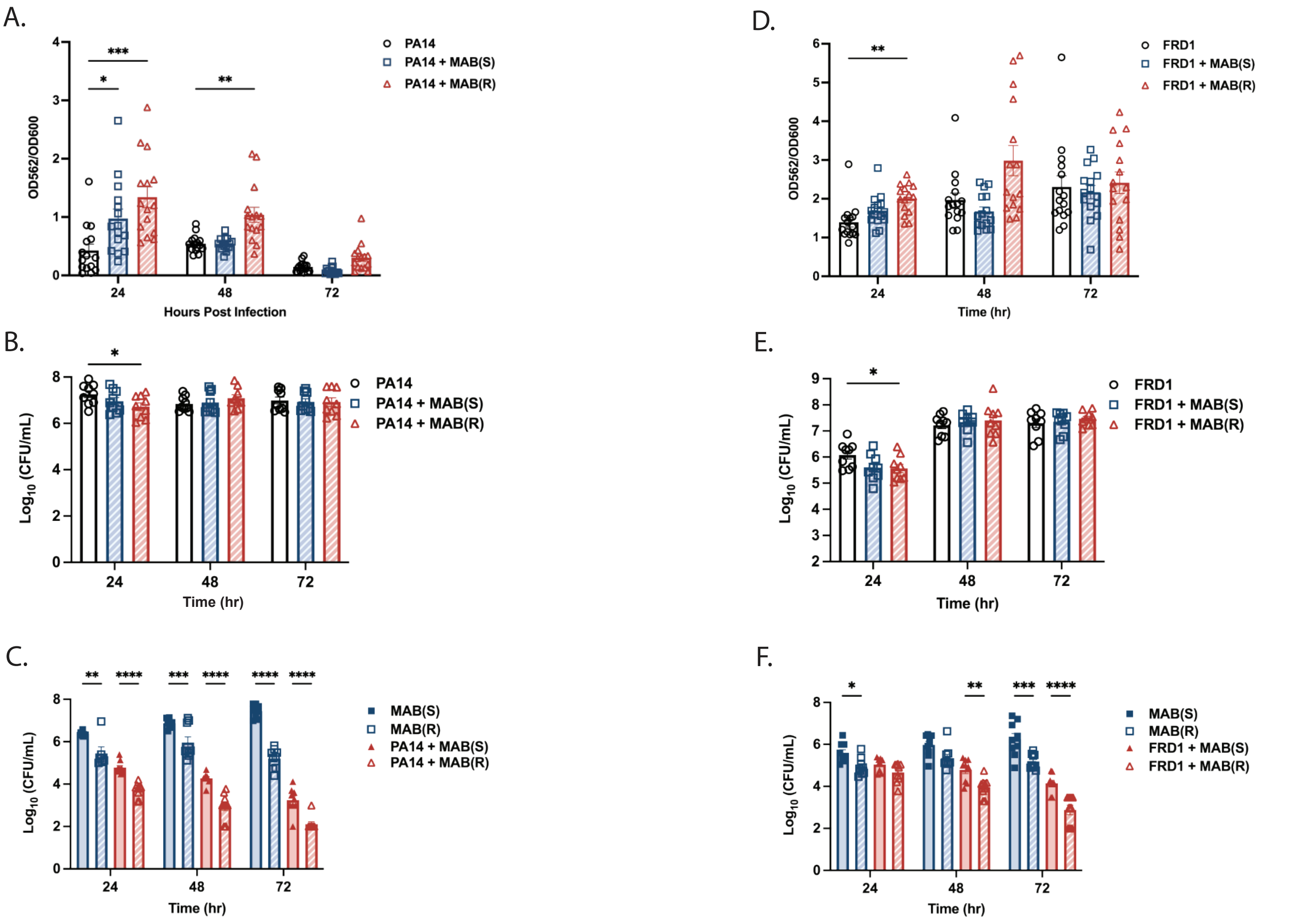

Supplementary Figure 7. Dual species biofilms with *P. aeruginosa* strains PA14 and FRD1
M. abscessus ATCC 19977 smooth and rough morphotypes were co-cultured with either of two *P. aeruginosa* strains (FRD1 or PA14) in LB medium in a 96-well plate for 24, 48, or 72 hours at 37 °C. Biofilm biomass was then measured using crystal violet staining for A) FRD1 or D) PA14 during single- and co-culture (n = 3 biological replicates, 6 technical). Two-way ANOVA with Tukey's multiple comparisons test. Viable colony forming units of *P. aeruginosa* B) FRD1 or E) PA14 during single- and co-culture, and of M. abscessus during single- and co-culture with C) FRD1 and F) PA14 were measured (n = 3 biological replicates, 3 technical). Two-way ANOVA with Tukey's multiple comparisons test.

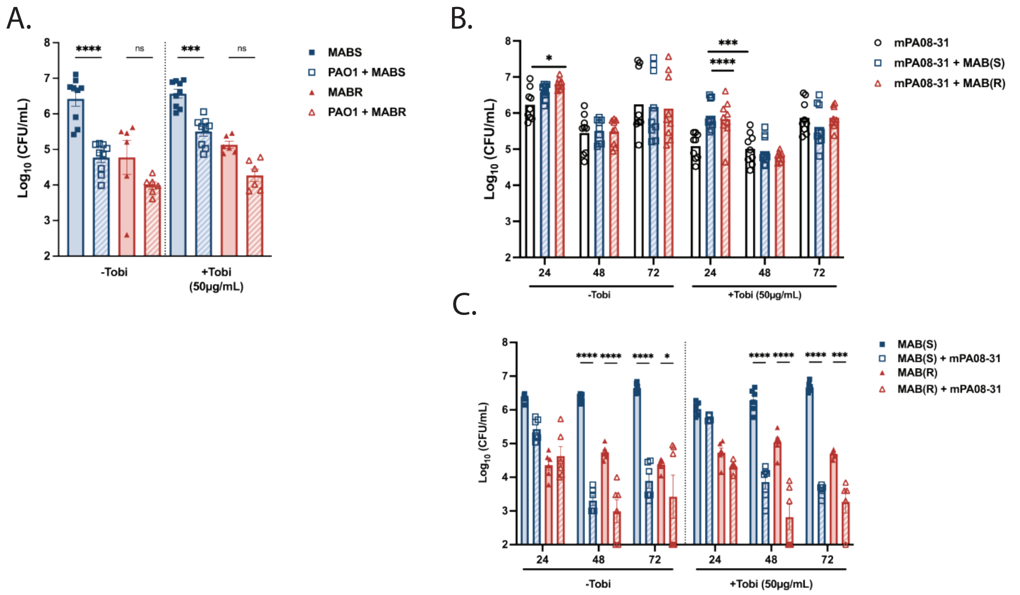

Supplementary Figure 8. Tobramycin treatment does not affect killing of MAB by *P. aeruginosa*
M. abscessus ATCC 19977 smooth and rough morphotypes were co-cultured with PAO1 or in LB medium in a 96-well plate for 24, 48, or 72 hours at 37 °C. Biofilms were then treated for 1 hour with tobramycin (50 µg/mL) or 1X PBS. A) Viable colony forming units of *M. abscessus* in single- or dual-species biofilms with *P. aeruginosa* PAO1 were measured. Viable colony counts of B) *P. aeruginosa* mPA08-31 and C) *M. abscessus* during single- and dual-species culture with and without tobramycin treatment were measured via differential plating at all time points. (n = 3 biological replicates, 3 technical). One-way ANOVA with Tukey’s multiple comparisons test.

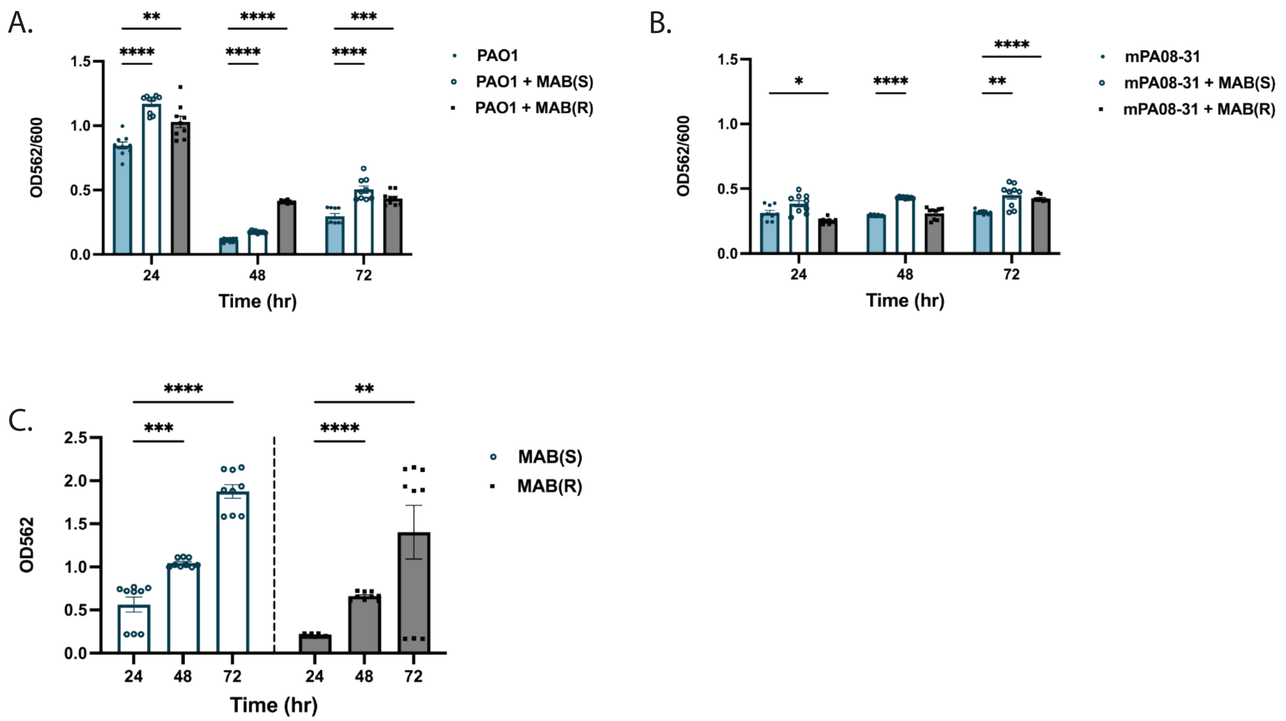

Supplementary Figure 9. Adaptation of experimental setup to a 6-well plate format for RNA-sequencing
M. abscessus ATCC 19977 smooth and rough morphotypes were co-cultured with either of two *P. aeruginosa* strains (PAO1 or mPA08-31) in LB medium in a 6-well plate for 24, 48, or 72 hours at 37 °C. Biofilm biomass was then measured using crystal violet staining for A) PAO1 or B) mPA08-31 during single- and co-culture. C) Development of single-species biofilms of *M. abscessus* over time as measured via crystal violet assay (OD562). Normalization was not used due to relatively low OD600 readings in comparison to P. aeruginosa. (n = 3 biological replicates, 3 technical). Two-way ANOVA with Tukey's multiple comparisons test.
cal). One-way ANOVA with Tukey's multiple comparisons test.

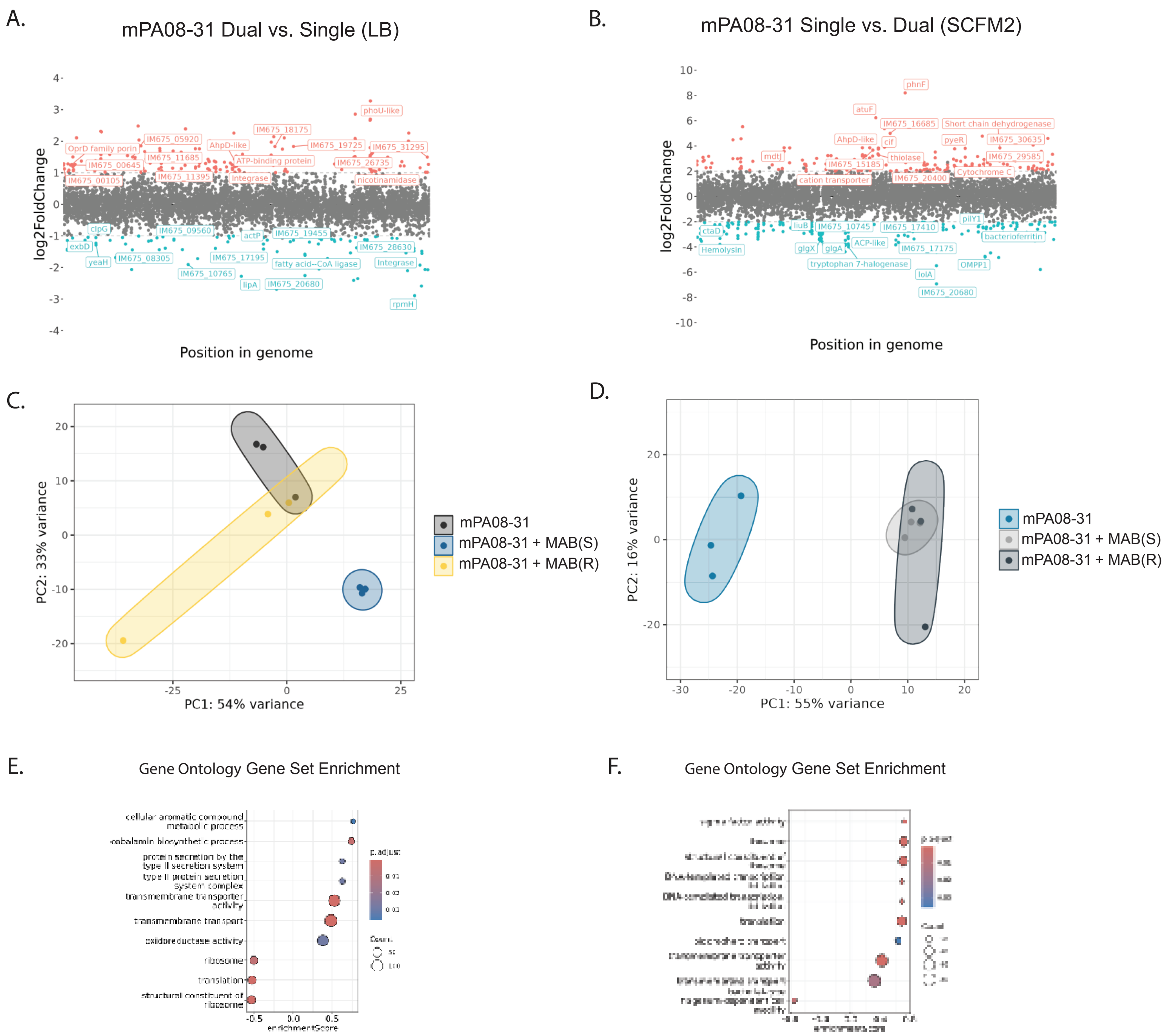

Supplementary Figure 10. RNA sequencing of *P. aeruginosa* mPA08-31 biofilms with and without MAB
M. abscessus ATCC 19977 smooth and rough morphotypes were co-cultured with *P. aeruginosa* mPA08-31 in LB medium or SCFM2 in a 6-well plate for 48 hours at 37 °C. Adherent cells were pooled (3 per sample) for RNA extraction and sequencing (n = 3). A,B) XY-plot of differentially expressed genes during mPA08-31 single or dual-species biofilms determined via differential expression analysis using DESeq2. B,C) Principal-component analysis of samples based on RNA sequencing data, colored by infection group. D,E) Most differentially expressed pathways as determined by Gene Ontology gene set enrichment analysis.

Supplemental Data 1. Gene counts mapped to *P. aeruginosa* PAO1 in RNA-sequencing experiments

Supplemental Data 2. Gene counts mapped to *P. aeruginosa* mPA08-31 in RNA-sequencing experiments

Supplemental Data 3. Differential expression of genes from *P. aeruginosa* PAO1 in single- and dual-species biofilms in LB

Supplemental Data 4. Differential expression of genes from *P. aeruginosa* PAO1 in single- and dual-species biofilms in SCFM2

Supplemental Data 5. Differential expression of genes from *P. aeruginosa* mPA08-31 in single- and dual-species biofilms in LB

Supplemental Data 6. Differential expression of genes from *P. aeruginosa* mPA08-31 in single- and dual-species biofilms in SCFM2

Supplemental Data 7. Supplemental statistical details
